# GABAB-Dependent Negative Feedback Contributes to Earlier Epileptiform Discharge Termination Within Malformed Cortex

**DOI:** 10.3390/ijms27177896

**Published:** 2026-09-04

**Authors:** Dmitry V. Amakhin, Elena B. Soboleva, Anna A. Kovalenko, Tatiana Yu. Postnikova, Aleksey V. Zaitsev

**Affiliations:** Laboratory of Molecular Mechanisms of Neural Interactions, Sechenov Institute of Evolutionary Physiology and Biochemistry of RAS, 194223 Saint Petersburg, Russia; dmitry.amakhin@gmail.com (D.V.A.); soboleva.elena.1707@gmail.com (E.B.S.); kovalenko_0911@mail.ru (A.A.K.); tapost2@mail.ru (T.Y.P.)

**Keywords:** focal cortical malformation, GABA_B_ receptors, epileptiform discharges, discharge termination, slow afterhyperpolarization, postsynaptic potassium current, negative feedback, freeze-induced microgyria

## Abstract

Malformations of cortical development frequently underlie drug-resistant epilepsy, yet little is known about how malformed cortical networks terminate epileptiform activity. In acute cortical slices from juvenile male Wistar rats with a focal freeze lesion, we compared the microgyrus and paramicrogyral zone during low-Mg^2+^/gabazine-induced epileptiform activity. Discharges terminated earlier in the microgyrus than in the paramicrogyral zone (median, 268 vs. 496 ms), without detectable regional differences in peak discharge-associated inward current or the weighted decay time constant of extracellular K^+^ transients. In separate voltage-clamp recordings, the transition to a slow post-discharge outward current occurred earlier in microgyral neurons, and the current peaked sooner and showed a smaller normalized late component. GABA_B_ receptor blockade with CGP-55845 prolonged discharges and preferentially disrupted the faster post-peak current decay in the microgyrus. Intracellular QX-314, used to probe a postsynaptic component, eliminated detectable regional differences in outward-current kinetics. *Gabbr1* and *Gabbr2* mRNA abundance did not differ detectably between the microgyrus and contralateral cortex. Overall, the findings support a postsynaptic GABA_B_-dependent contribution to earlier epileptiform discharge termination within the microgyrus. More broadly, malformation-associated reorganization includes local negative-feedback processes that constrain pathological network persistence alongside mechanisms that promote hyperexcitability.

## 1. Introduction

Malformations of cortical development are important causes of epilepsy and neurodevelopmental impairment, often presenting in childhood [1,2]. Experimental models have been used to study how abnormal cortical development alters neuronal excitability and local circuit organization. In the rat focal freeze lesion (FFL) model, neonatal cortical injury produces a three- to four-layered microgyrus [3,4]. The surrounding paramicrogyral zone (PMZ) retains a six-layered organization but undergoes substantial functional reorganization [4,5,6]. The microgyrus and PMZ thus represent distinct regions within the same affected cortical network.

Research on the FFL model has largely addressed the mechanisms of cortical hyperexcitability. The PMZ is particularly susceptible to epileptiform activity, and changes in excitatory and inhibitory synaptic connectivity have been described in both pyramidal neurons and fast-spiking interneurons [5,6,7,8]. Altered expression of glutamate and GABA receptors and chloride transporters has also been reported within and around the malformation [9,10,11,12]. We previously found that most basic intrinsic membrane properties of layer 2/3 pyramidal neurons adjacent to the microgyrus were preserved, whereas synaptic integration and the balance between excitation and inhibition were shifted toward excitation [13]. However, the cellular mechanisms governing epileptiform discharge duration and termination across different regions of the FFL cortex remain poorly defined.

Epileptiform discharge termination may involve more than a gradual decline in excitatory drive. Intense neuronal activity changes intra- and extracellular ion concentrations and recruits electrogenic ion transport, processes that can shape both discharge termination and the subsequent period of reduced excitability [14,15,16,17]. Because these processes operate over different time scales, regional differences in their recruitment could influence discharge duration. In our previous study, discharges induced by reduced extracellular Mg^2+^ and GABA_A_ receptor blockade were more frequent but shorter in FFL-containing slices than in slices from sham-operated animals, while no difference was detected in the peak amplitude of discharge-associated inward currents [13]. That study did not establish which region accounted for the shorter discharge duration or identify the underlying cellular mechanism.

GABA_B_ receptor signaling is one possible source of slow inhibitory feedback. GABA_B_ receptors regulate neurotransmitter release presynaptically and activate potassium conductances postsynaptically [18,19,20]. Their effects on epileptiform activity are context-dependent. GABA_B_ receptor antagonists have prolonged or potentiated epileptiform events in hippocampal and neocortical slices [21,22], whereas activation of presynaptic GABA_B_ receptors can reduce glutamate release and contribute to the effects of low-frequency stimulation [23]. The resulting network response depends on the preparation and on the relative involvement of pre- and postsynaptic receptors [24]. In the FFL model, GABA_B_ receptors have been examined at the level of receptor binding [9], whereas studies of inhibitory alterations have mainly addressed GABA_A_-related synaptic, receptor-expression, and chloride-dependent mechanisms [8,11,12]. Whether slow GABA_B_-dependent feedback differs between the microgyrus and PMZ has not been established.

Here, we first compared epileptiform discharge termination in simultaneous recordings from the microgyrus and PMZ. We then characterized a slow outward current that emerged as the discharge-associated inward current subsided and examined its possible contribution to the regional difference in termination. Extracellular K^+^ recordings were used to assess regional ion dynamics. The involvement of GABA_B_ signaling was investigated using receptor blockade at the network level and intracellular QX-314 to assess a postsynaptic QX-314-sensitive component. As a targeted molecular follow-up, we quantified *Gabbr1* and *Gabbr2* mRNA in the microgyrus and the corresponding region of the contralateral cortex.

## 2. Results

### 2.1. Earlier Epileptiform Discharge Termination in the Microgyrus

**Figure 1:**
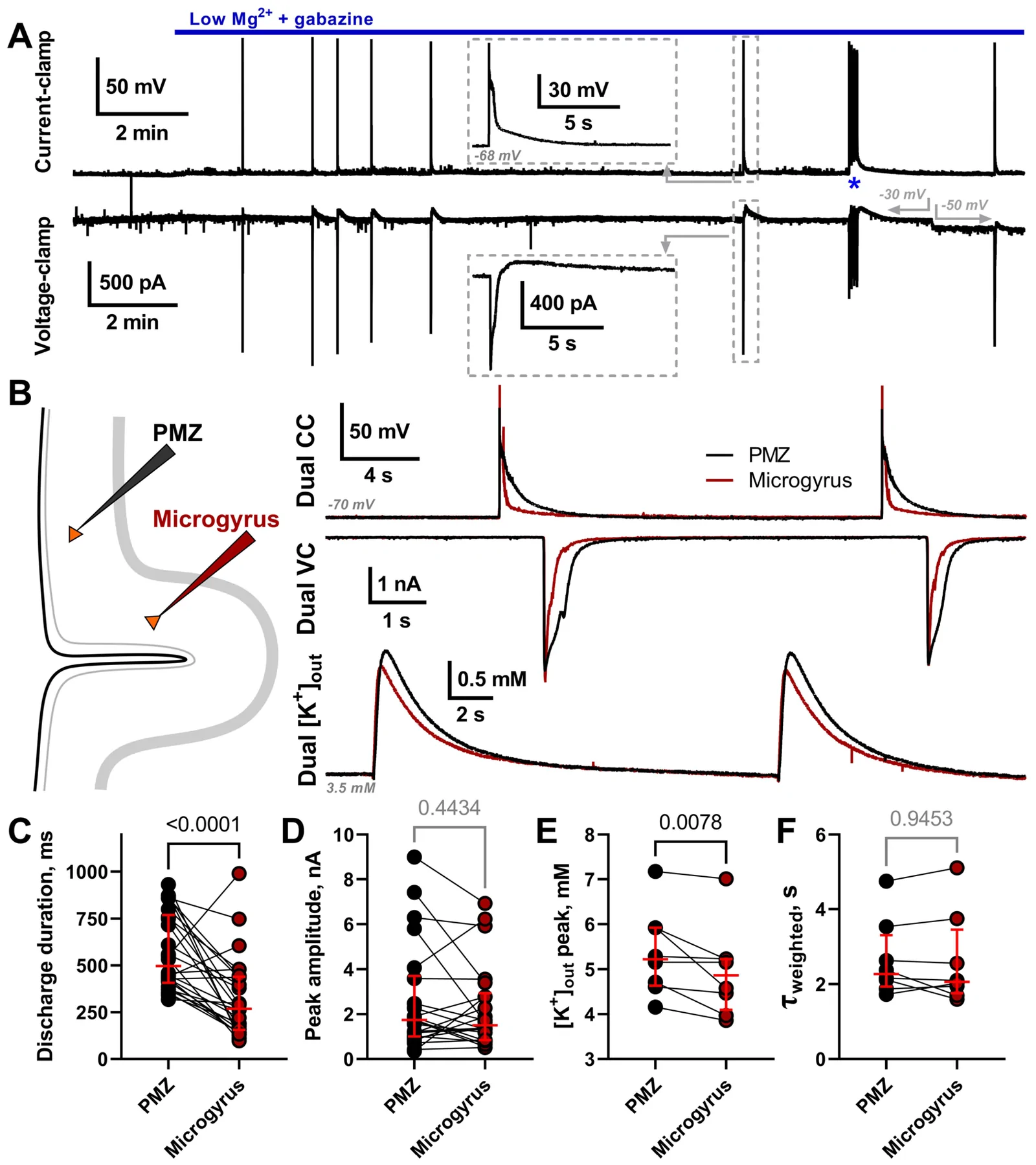
Comparative properties of epileptiform discharges in the microgyrus and PMZ. (**A**) Simultaneous dual recordings under low-Mg^2+^/gabazine conditions. The blue asterisk marks a high-frequency burst of epileptiform discharges that was excluded from the quantitative analyses. (**B**) Recording configuration (left) and representative simultaneous recordings from the PMZ and microgyrus (right). Black, PMZ; red, microgyrus. (**C**–**F**) Discharge duration (**C**; *n* = 26 pairs, N = 13), peak discharge-associated inward-current amplitude (**D**; *n* = 22 pairs, N = 11), peak [K^+^]_o_ transient (**E**; *n* = 8 pairs, N = 4), and weighted decay time constant of the [K^+^]_o_ transient (**F**; *n* = 8 pairs, N = 4). Wilcoxon signed-rank test.

Epileptiform activity was induced by perfusing brain slices with artificial cerebrospinal fluid (ACSF) containing a reduced Mg^2+^ concentration (0.25 mM) and the GABA_A_ receptor antagonist gabazine (20 µM). Under these conditions, slices generated stereotypical epileptiform discharges at a frequency of 0.25–2.5 events per minute (Figure 1A). Occasional high-frequency bursts comprising 2–7 discharges, prolonged seizure-like events, and spreading depolarizations were excluded from the analyses reported below (these forms of activity were described in our previous study). A total of nine paired recordings were completely excluded because spreading depolarization occurred within the analysis window (*n* = 7), or because high-frequency bursts (*n* = 1) or seizure-like events (*n* = 1), rather than regular stereotypical discharges, predominated.

To compare discharge properties between the two regions, we performed paired recordings with one electrode positioned within the microgyrus and the other in layer II/III of the PMZ, approximately 200 µm from the microgyral border (Figure 1B). Neuronal activity was assessed using dual whole-cell patch-clamp recordings or simultaneous whole-cell and extracellular potassium concentration ([K^+^]_o_) recordings with ion-selective microelectrodes. In all analyzed dual recordings, epileptiform discharges occurred synchronously at both sites (Figure 1B). Recordings were obtained from N = 13 animals for duration measurements, N = 11 animals for current amplitude measurements, and N = 4 animals for [K^+^]_o_ measurements.

In paired current-clamp recordings, discharges were significantly shorter within the microgyrus than in simultaneously recorded PMZ neurons (microgyrus: median 268 ms [IQR: 155–439]; PMZ: median 496 ms [IQR: 408–769]; *n* = 26 pairs; *p* < 0.0001, Wilcoxon signed-rank test; Figure 1C). To test whether the shorter duration was accompanied by a smaller peak discharge-associated inward current, we measured the peak current at a holding potential of −50 mV in paired voltage-clamp recordings. No difference in peak amplitude was detected between the PMZ and microgyrus (PMZ: median 1.74 nA [IQR: 1.00–3.70]; microgyrus: median 1.51 nA [IQR: 0.84–2.93]; *n* = 22 pairs; *p* = 0.44, Wilcoxon signed-rank test; Figure 1D).

We next examined whether regional differences in extracellular K^+^ dynamics could account for the shorter discharges. Paired [K^+^]_o_ recordings revealed a higher peak [K^+^]_o_ transient in the PMZ than in the microgyrus (PMZ: median 5.2 mM [IQR: 4.6–5.9]; microgyrus: median 4.9 mM [IQR: 4.1–5.2]; *n* = 8 pairs; *p* = 0.008, Wilcoxon signed-rank test; Figure 1E), accompanying the longer discharge duration in the PMZ. However, no difference was detected in the weighted decay time constant of the [K^+^]_o_ transient (PMZ: median 2.3 s [IQR: 1.9–3.3]; microgyrus: median 2.1 s [IQR: 1.8–3.5]; *n* = 8 pairs; *p* = 0.95, Wilcoxon signed-rank test; Figure 1F), providing no evidence for faster decay of the measured extracellular K^+^ signal within the microgyrus.

**Figure A1:**
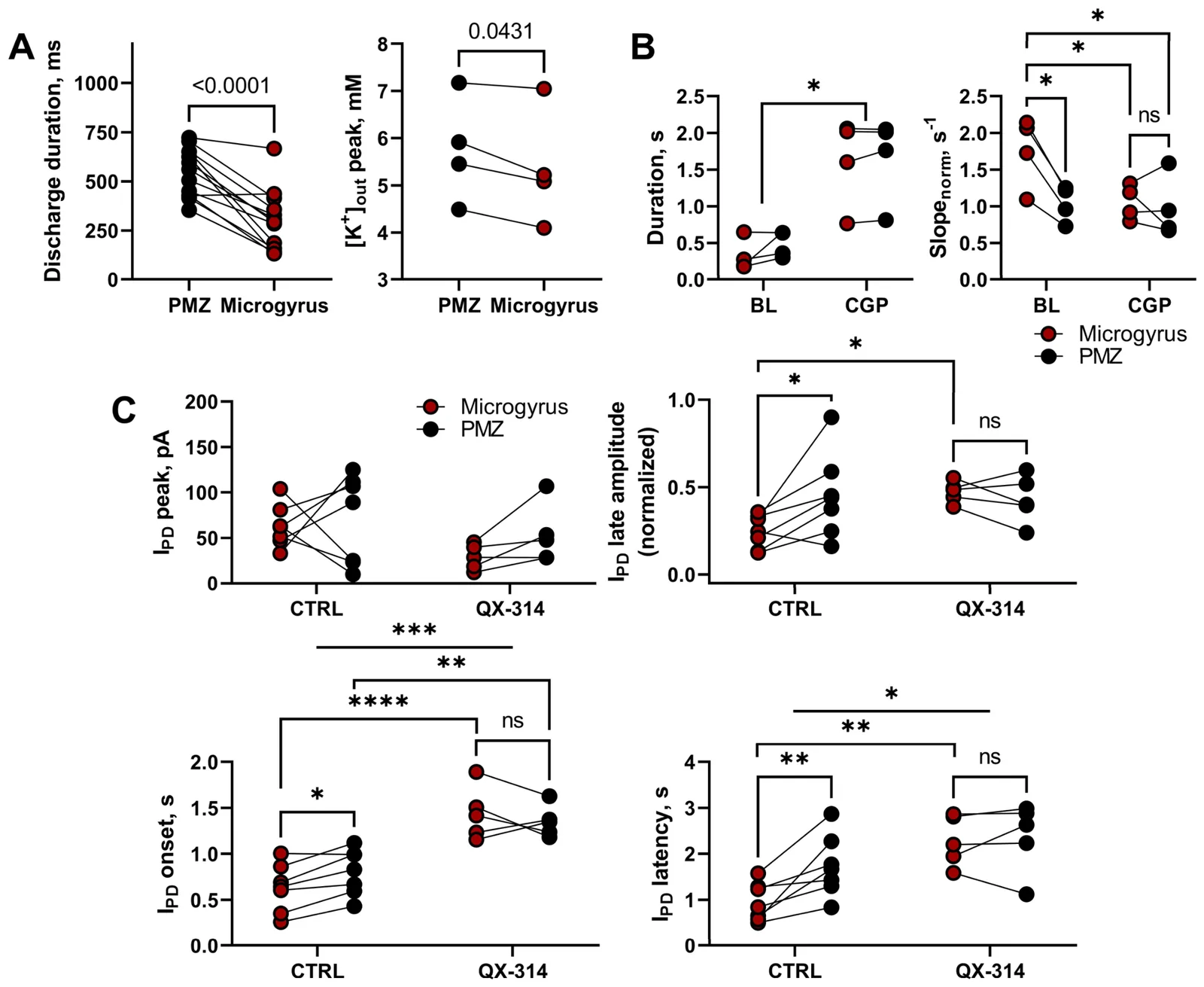
(**A**) Microgyrus vs. PMZ discharge duration (left, N = 13 animals; paired *t*-test) and peak [K^+^]_o_ transient (right, N = 4 animals; paired *t*-test). (**B**) Evoked discharge duration (left, two-way repeated-measures ANOVA; F(1,3) = 28.2, *p*(CGP) = 0.013) and normalized current decay slopes (right; two-way repeated-measures ANOVA; F(1,3) = 11.9, *p*(CGP × location) = 0.041). (**C**) *I*_PD_ peak amplitude, normalized late amplitude, zero-crossing time, and peak latency under standard vs. QX-314-containing intracellular conditions (N = 7 and N = 5 animals, respectively; mixed-model ANOVA). * *p* < 0.05, ** *p* < 0.01, *** *p* < 0.001, **** *p* < 0.0001; ns, not significant; Sidak’s post hoc test. See Table A3 for full ANOVA results.

To account for potential non-independence of observations obtained from the same animal, the key comparisons reported in this section were reanalyzed using an animal-level summary approach (Appendix B, Figure A1A). This analysis confirmed the significant difference in discharge duration and [K^+^]_o_ peak.

Thus, epileptiform discharges terminated earlier within the microgyrus than in the PMZ. This regional difference was not accompanied by a detectable reduction in peak discharge-associated inward current or by faster decay of the measured extracellular K^+^ transient, suggesting that neither of these measured parameters is sufficient to explain the earlier discharge termination within the microgyrus.

### 2.2. Complex Afterpotentials Follow Epileptiform Discharges

**Figure 2:**
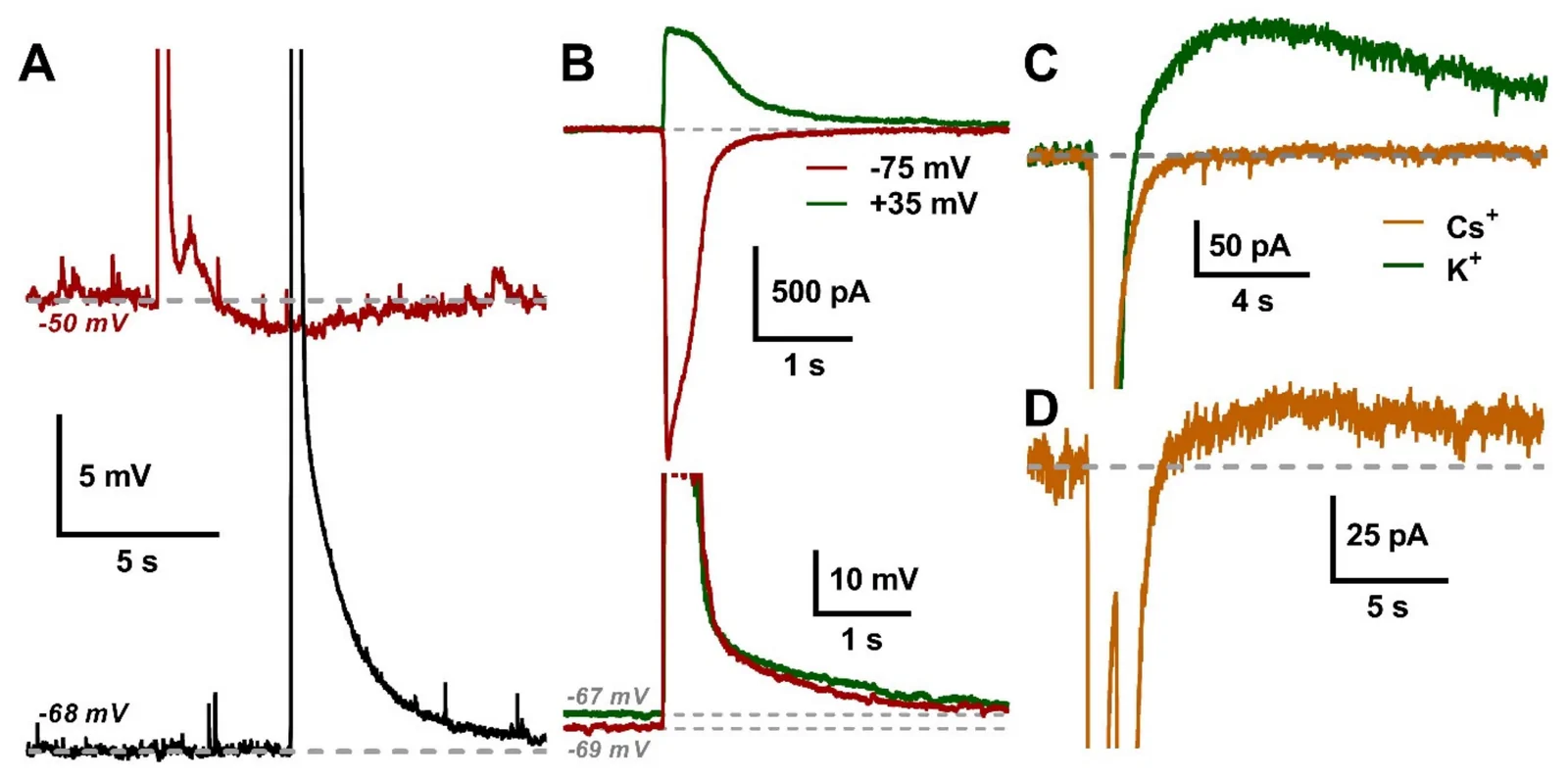
Characterization of post-discharge afterpotentials and outward currents in neocortical neurons. All representative traces were obtained from sham-operated control slices. (**A**) Current-clamp recordings from the same neuron showing epileptiform discharges followed by an sADP near resting membrane potential (black trace) and an sAHP during membrane depolarization produced by steady current injection (red trace). (**B**) Dual recordings during two color-coded epileptiform discharges. Top: voltage-clamp recordings from one neuron held at −75 mV (red) or +35 mV (green). Bottom: simultaneous current-clamp recordings from a neighboring neuron. At −75 mV, the discharge-associated synaptic current returns toward baseline before the sADP decays. (**C**) Simultaneous voltage-clamp recordings from two neurons held at −20 mV using K^+^-based (green) and Cs^+^-based (orange) intracellular solutions. After an isolated discharge, a clear $I_{PD}$ is present under the K^+^-based but not the Cs^+^-based condition. (**D**) A high-frequency burst evokes a small residual $I_{PD}$ under the Cs^+^-based condition.

Whole-cell current-clamp recordings revealed that epileptiform discharges were followed by complex afterpotentials in neurons from sham-operated control slices and from the PMZ of FFL-containing slices. These afterpotentials comprised depolarizing and hyperpolarizing components, whose relative prominence depended on membrane potential. Representative recordings from sham-operated control slices are shown in Figure 2.

At resting or hyperpolarized membrane potentials, a slow afterdepolarization (sADP) typically followed each discharge. In a subset of recordings (4 of 20 current-clamp recordings in sham-operated control slices and 5 of 30 recordings in the PMZ) the sADP gradually transitioned into a prominent slow afterhyperpolarization (sAHP) lasting more than 6 s. When neurons were depolarized by constant current injection, the sADP was largely replaced by the sAHP (Figure 2A). Consistent with these observations, voltage-clamp recordings using a K^+^-gluconate-based pipette solution (solution 2) revealed a post-discharge outward current (*I*_PD_) at depolarized holding potentials (Figure 1A, bottom trace).

To assess whether the sADP at resting potential reflected a persistent synaptic current, we performed dual recordings with one neuron in current-clamp mode (K^+^-gluconate-based solution 1) and a neighboring neuron in voltage-clamp mode (Cs^+^-methanesulfonate-based solution 3). At a holding potential of −75 mV, the discharge-associated synaptic current returned toward baseline before the sADP had decayed in recordings from both sham-operated control slices (*n* = 5) and the PMZ (*n* = 6; Figure 2B), indicating that a persistent synaptic current was unlikely to be the sole source of the sADP. At +35 mV, the discharge-associated current decayed more slowly, consistent with an increased contribution of NMDA receptor-mediated current (Figure 2B).

The *I*_PD_ observed with K^+^-based pipette solution was absent when using Cs^+^-based solution (Figure 2C; dual voltage-clamp recording at −20 mV). However, following high-frequency burst of discharges (observed in 3 out of 8 recordings in control slices and 5 out of 12 recordings in PMZ), a small residual *I*_PD_ persisted even with Cs^+^ (Figure 2D), indicating that the *I*_PD_ is predominantly mediated by Cs^+^-sensitive K channels, with a minor component attributable to Cs^+^-insensitive mechanisms [17,25].

**Figure 3:**
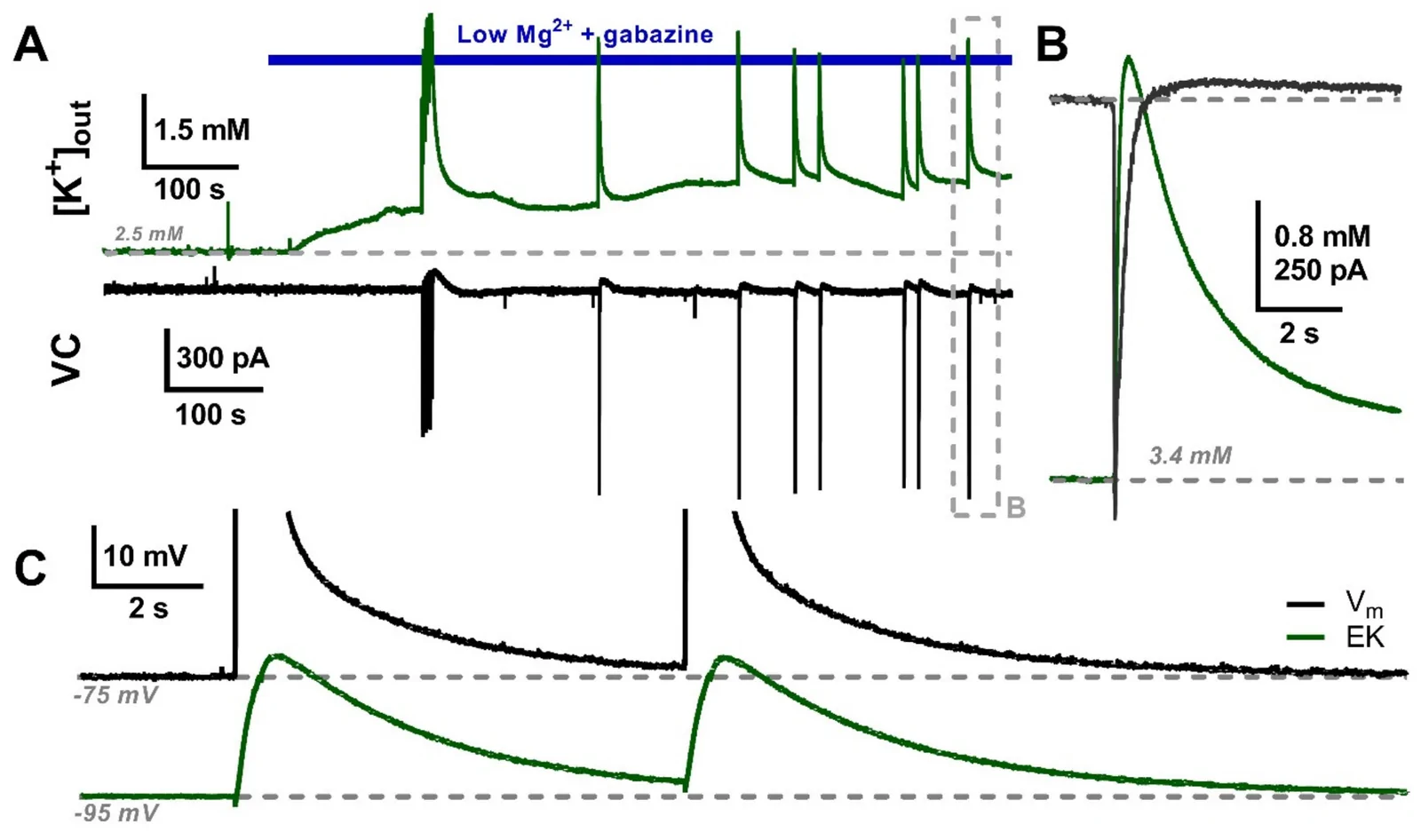
Extracellular potassium dynamics associated with the post-discharge slow afterdepolarization (sADP). (**A**) Representative simultaneous recording of extracellular potassium concentration ([K^+^]_o_, top trace) and neuronal membrane current in voltage-clamp mode (bottom trace, V_h_ = −30 mV) during perfusion with low-Mg^2+^/gabazine solution (blue bar). The boxed region is expanded in B. (**B**) Expanded view showing that [K^+^]_o_ rises during an epileptiform discharge and returns toward baseline over several seconds. (**C**) Representative simultaneous recording of neuronal membrane potential in current-clamp mode (V_m_, black trace) and the K^+^ equilibrium potential (E_K_, green trace) calculated from the [K^+^]_o_ recording using the Nernst equation. The calculated E_K_ and the sADP show similar post-discharge decay time courses.

Because the sAHP was most clearly revealed at depolarized membrane potentials and was commonly obscured by the sADP near rest, we next examined the possible origin of the sADP. Since membrane potential is influenced by the K^+^ gradient, we performed simultaneous whole-cell and extracellular K^+^ concentration ([K^+^]_o_) recordings using ion-selective microelectrodes (Figure 3A). These recordings revealed transient elevations of [K^+^]_o_ by several millimolar during epileptiform discharges. The [K^+^]_o_ increase coincided with the discharge, whereas its return toward baseline continued for several seconds after discharge termination (Figure 3B). The resulting depolarizing shift in the calculated K^+^ equilibrium potential (E_K_) would reduce the outward K^+^ driving force and was temporally consistent with a contribution to the post-discharge sADP. In simultaneous recordings of [K^+^]_o_ and membrane potential, the calculated E_K_ and the sADP exhibited similar post-discharge decay time courses (Figure 3C; Equation (2)).

### 2.3. GABA_B_ Receptors Contribute to Epileptiform Discharge Termination

Our finding that discharges terminate significantly faster within the microgyrus suggests the presence of an enhanced negative feedback mechanism in this region. Given that GABA_B_ receptor activation generates slow inhibitory currents in neocortical neurons [18,19,20] and contributes to seizure termination [22,24], we hypothesized that altered GABA_B_ receptor-mediated inhibition might underlie the accelerated discharge cessation observed in the microgyrus.

**Figure 4:**
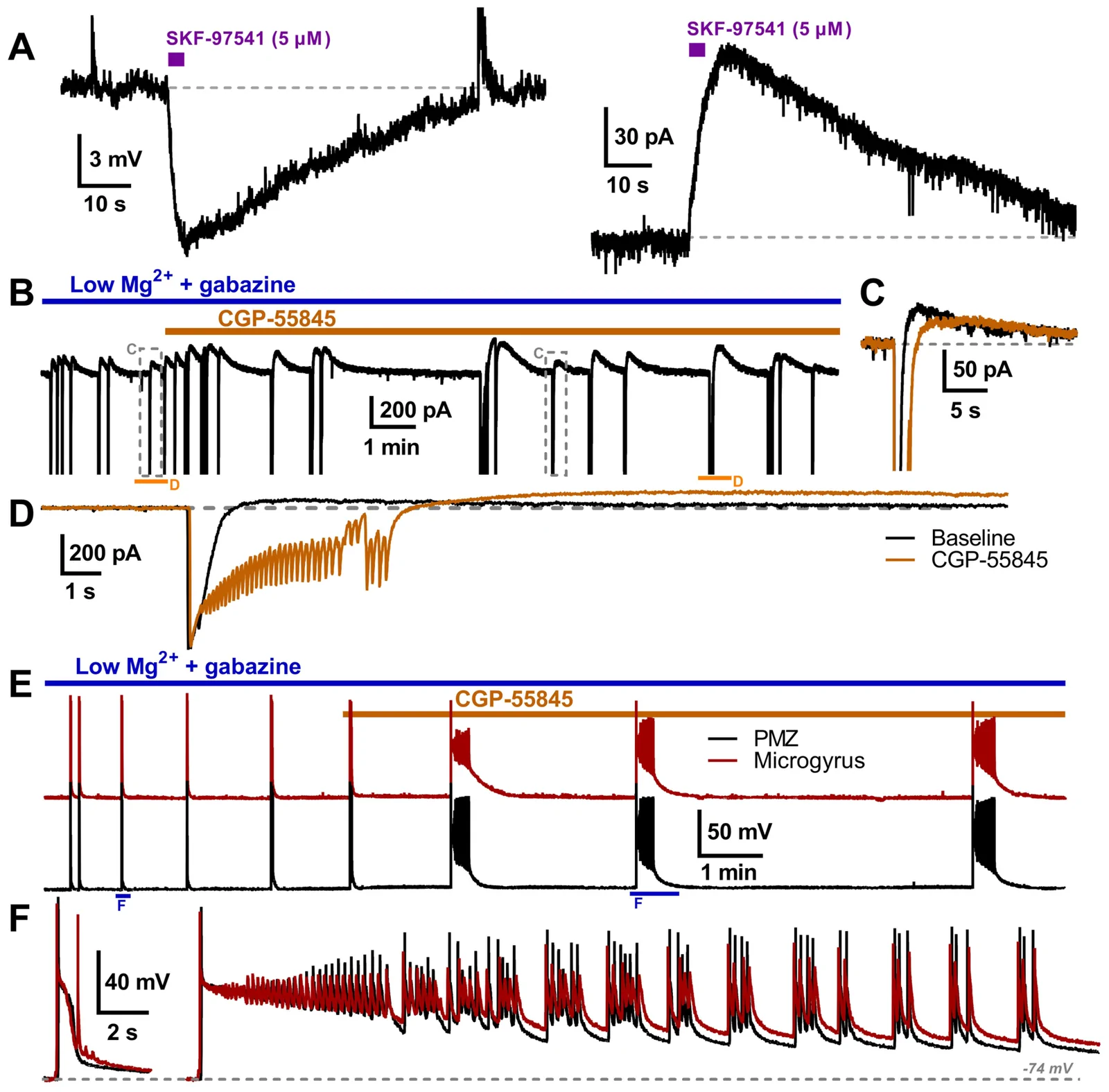
GABA_B_ receptor modulation affects epileptiform discharge duration in control and malformed cortex. (**A**) Local application of the GABA_B_ receptor agonist SKF-97541 (5 µM, 3 s) induces a slow hyperpolarization in current-clamp (left, pipette solution 1) and an outward current in voltage-clamp (right, pipette solution 2; *n* = 5 cells from N = 2 animals). (**B**) Bath application of CGP-55845 (3 µM) transforms brief discharges into prolonged multi-peak events. (**C**) Expanded view from (**B**) (gray boxes). CGP-55845 prolongs discharge duration and alters the *I*_PD_. (**D**) Expanded view from (**B**) (orange lines) showing comparable initiation but divergent termination. Panels (**B**–**D**) are representative of *n* = 15 recordings from N = 5 control animals. (**E**) CGP-55845 transforms spontaneous activity in the FFL slice (recording configuration as in Figure 1B). Blue lines mark regions expanded in (**F**). (**F**) Expanded view illustrating transformation to prolonged ictal-like events; *n* = 12 recordings from N = 4 FFL animals.

We first confirmed the presence of functional postsynaptic GABA_B_ receptors on layer 2/3 pyramidal neurons of control animals. Local application of the GABA_B_ receptor agonist SKF-97541 (5 µM) to the recorded neuron elicited a slow hyperpolarization with an amplitude of 8 ± 1 mV (*n* = 5, N = 2) at a membrane potential of −70 mV in current-clamp mode, and a corresponding outward current in voltage-clamp mode (Figure 4A).

We next examined the effect of GABA_B_ receptor blockade on network activity. Bath application of the selective antagonist CGP-55845 (3 µM) to control slices altered network activity, transforming brief, stereotypical discharges into prolonged events of variable duration featuring multiple current peaks (*n* = 15 recordings, N = 5; Figure 4B–D). While CGP-55845 did not abolish the *I*_PD_ in whole-cell recordings, this shift in network pattern precluded a direct comparison of *I*_PD_ properties before and after drug application, as both the triggering stimulus and underlying *I*_PD_ mechanisms may have been altered. For discharges of comparable duration, the *I*_PD_ peak amplitude appeared reduced in the presence of CGP-55845 (Figure 4C).

We confirmed that CGP-55845 also prolonged discharges in FFL slices, both within and outside the microgyrus (Figure 4E,F). Similar to control slices, the antagonist transformed brief discharges into prolonged multi-peaked events across all recordings, specifically extending the late discharge phase without altering initiation. The ictal-like discharges induced by CGP-55845 in FFL slices were longer than those in control slices (control: 5.9 ± 2.3 s, *n* = 11, N = 5; FFL: 11.0 ± 6.8 s, *n* = 12, N = 4; *p* = 0.029, Welch’s *t*-test).

**Figure 5:**
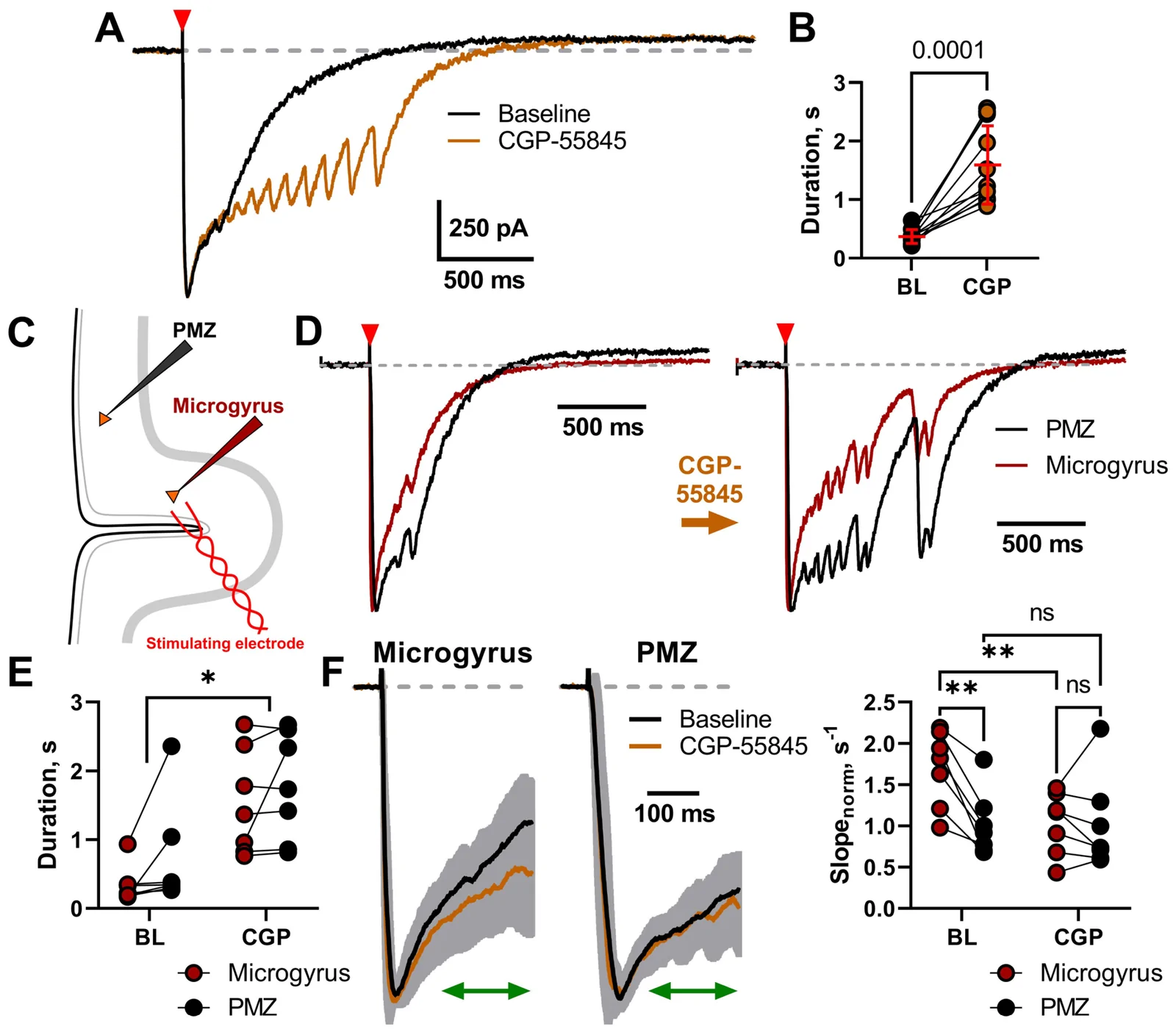
The effect of GABA_B_ receptor blockade on evoked epileptiform discharges. (**A**) Voltage-clamp recordings in control slices before and after CGP-55845 application (Vh = −20 mV). (**B**) Discharge duration before and after CGP-55845 application in control slices (*n* = 11 paired recordings, N = 5; paired *t*-test). (**C**) Schematic showing the stimulating electrode within the microgyrus and recording pipettes positioned in the microgyrus and PMZ. (**D**) Superimposed recordings from both locations before and after CGP-55845 (normalized to peak). (**E**) Statistical comparison of evoked discharge duration (two-way repeated-measures ANOVA, *n* = 7 pairs, N = 4). (**F**) Left: averaged recordings (normalized, Vh = −20 mV); gray area represents SD. Right: normalized current decay slopes (Slopenorm; two-way repeated-measures ANOVA, *n* = 7 pairs, N = 4). * *p* < 0.05, ** *p* < 0.01; ns, not significant; Sidak’s post hoc test.

Since bath application of CGP-55845 altered the overall properties of spontaneous epileptiform activity, resulting in discharges of variable duration, we employed local extracellular stimulation to standardize the triggering stimulus and quantify discharge termination dynamics (Figure 5). In control slices, a bipolar stimulating electrode was placed near the recorded neuron. In FFL slices, the stimulating electrode was positioned within the microgyrus, while simultaneous whole-cell recordings were obtained from two neurons: one within the microgyrus and one in the PMZ (Figure 5C). A single pulse (250–350 µA) evoked stereotypical discharges, allowing consistent quantification of discharge-associated inward currents at a holding potential of −20 mV.

In control slices, stimulus-evoked discharges initiated similarly before and after CGP-55845 application but exhibited prolonged termination (Figure 5A). Under baseline conditions, the evoked current displayed a single peak followed by a decay. After GABA_B_ receptor blockade, the current duration increased and secondary peaks emerged. Paired *t*-test analysis confirmed a significant prolongation of evoked discharge duration from 0.4 ± 0.1 s to 1.6 ± 0.7 s (Figure 5B; *p* = 0.0001, *n* = 11).

In FFL slices, CGP-55845 similarly prolonged evoked discharges both within and outside the microgyrus (Figure 5D). A two-way repeated measures ANOVA revealed a significant main effect of CGP-55845 application on discharge duration (F(1,6) = 10.4, *p* = 0.018), with no significant interaction between drug effect and neuronal localization (F(1,6) = 1.4, *p* = 0.3; Figure 5E). This result was confirmed using animal-level reanalysis (Appendix B, Figure A1B). This indicates that the antagonist prolonged discharges to a similar extent in both regions.

We next analyzed the kinetics of current decay by quantifying the normalized decay slope (Slopenorm) within a standardized post-peak window (70–220 ms after the initial peak; Figure 5F). A two-way repeated-measures ANOVA revealed a significant interaction between drug effect and neuron localization (F(1,6) = 12, *p* = 0.013). Under baseline conditions, normalized current decay was faster in the microgyrus than in the PMZ (microgyrus: 1.7 ± 0.5 s^−1^ vs. PMZ: 1.0 ± 0.4 s^−1^; Sidak’s post hoc test, *p* < 0.01). After CGP-55845 application, this regional difference was no longer detected (microgyrus: 1.0 ± 0.4 s^−1^ vs. PMZ: 1.0 ± 0.6 s^−1^; *p* > 0.05). Blockade slowed the decay slope in the microgyrus relative to baseline (*p* < 0.01), whereas no corresponding change was detected in the PMZ (*p* > 0.05). Animal-level reanalysis confirmed the drug-by-location interaction (Appendix B, Figure A1B).

Together, these results indicate that GABA_B_ receptor blockade prolongs evoked epileptiform discharges in both regions. Moreover, the significant drug-by-location interaction in normalized current decay shows that GABA_B_ receptor signaling preferentially contributes to the faster post-peak current decay observed within the microgyrus, although the data do not establish whether this contribution fully accounts for the shorter discharge duration in this region.

### 2.4. Intracellular QX-314 Eliminates Detectable Regional Differences in I*_PD_* Kinetics

Bath application of CGP-55845 affects both pre- and postsynaptic GABA_B_ receptors and therefore does not isolate the postsynaptic component within individual neurons. To probe a cell-restricted postsynaptic component, we used intracellular QX-314, which suppresses slow GABA_B_-associated K^+^ currents and can inhibit GIRK channels [26,27] but is not selective for GABA_B_ receptor-activated conductances.

**Figure 6:**
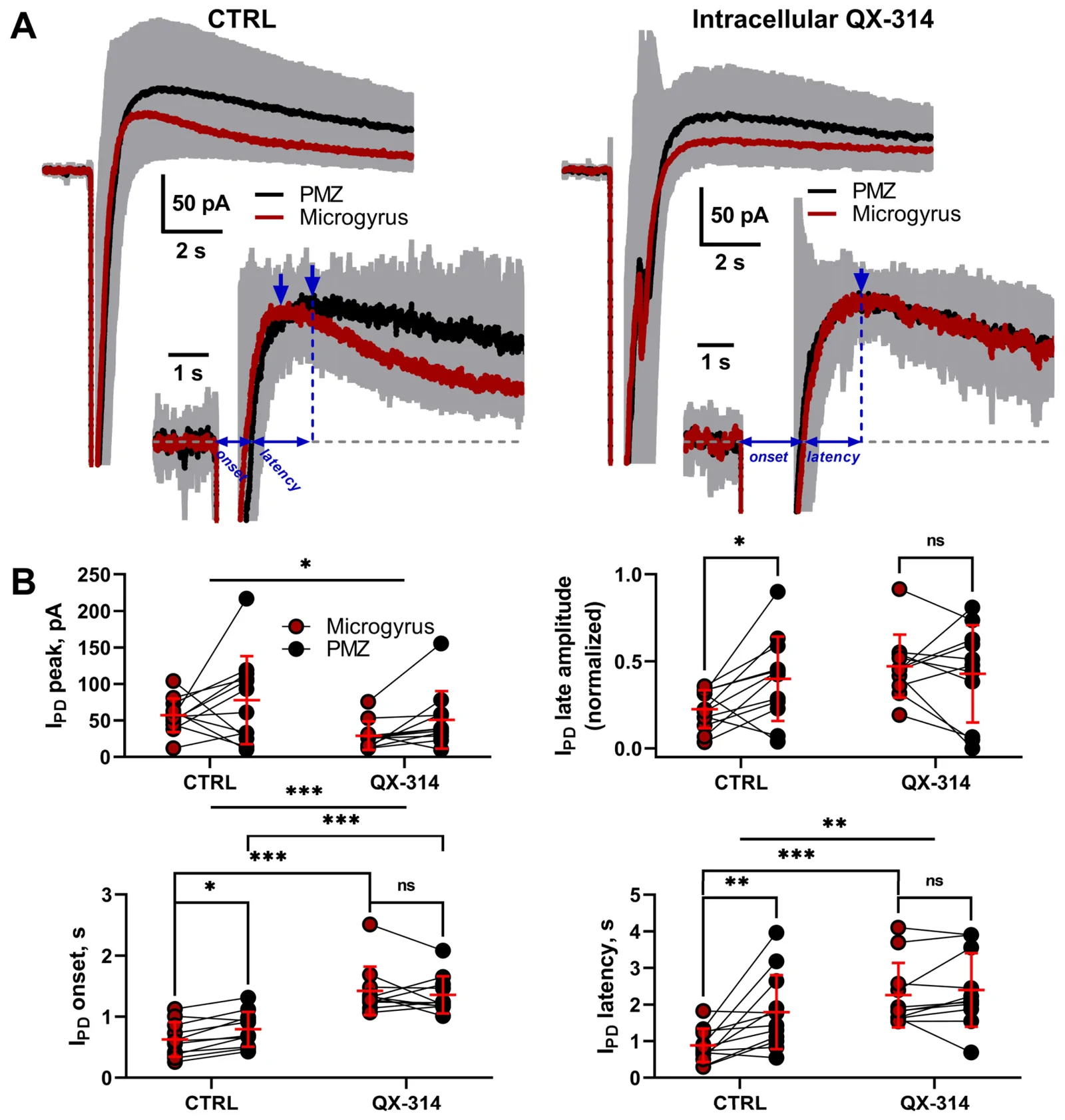
*I*_PD_ kinetics without and with intracellular QX-314 in FFL slices. (**A**) Averaged voltage-clamp recordings at −20 mV obtained using dual whole-cell recordings from paired microgyrus and PMZ neurons. Left, standard intracellular solution without QX-314 (*n* = 12 pairs, N = 7 animals); right, intracellular solution containing QX-314 (*n* = 11 pairs, N = 5 animals). Gray shading represents SD. Insets show the same recordings normalized to the peak. Blue arrows mark the peak, and the blue horizontal arrows illustrate the zero-crossing time and peak latency. (**B**) Paired regional values for *I*_PD_ peak amplitude, normalized late amplitude measured 10 s after the zero crossing, zero-crossing time, and peak latency. Intracellular solution was analyzed as a between-pairs factor and location as a within-pair factor using mixed-model ANOVA followed by Sidak’s post hoc test. Red, microgyrus; black, PMZ. * *p* < 0.05, ** *p* < 0.01, *** *p* < 0.001; ns, not significant. See Table A1 for full ANOVA results.

We performed dual voltage-clamp recordings in FFL slices from neuron pairs comprising one neuron within the microgyrus and one neuron in the PMZ, using either a standard pipette solution (solution 2) or a solution containing QX-314 (solution 4). Both neurons were held at −20 mV, where the discharge-associated inward current transitions into the outward $I_{PD}$, permitting continuous kinetic analysis (Figure 6A).

We quantified four $I_{PD}$ parameters: peak amplitude, zero-crossing time (termed $I_{PD}$ onset in Figure 6), peak latency measured from the zero crossing to the maximum outward current, and normalized late amplitude measured 10 s after the zero crossing and normalized to the peak. Mixed-model ANOVA was performed with intracellular solution as a between-pairs factor and neuronal location as a within-pair factor (Table A1; Figure 6B). Significant solution-by-location interactions were detected for zero-crossing time, peak latency, and normalized late amplitude, indicating that the effect of neuronal location on these parameters differed between the standard and QX-314-containing intracellular conditions.

Without intracellular QX-314, microgyral neurons exhibited an earlier $I_{PD}$ zero crossing than PMZ neurons (microgyrus: 0.63 ± 0.28 s; PMZ: 0.79 ± 0.28 s; Sidak’s post hoc test, *p* < 0.05). Peak latency was also shorter in the microgyrus (0.88 ± 0.46 s) than in the PMZ (1.79 ± 1.01 s; *p* < 0.01), and the normalized late amplitude was lower in the microgyrus (0.22 ± 0.11) than in the PMZ (0.40 ± 0.24; *p* < 0.05).

With intracellular QX-314, no regional differences were detected in zero-crossing time, peak latency, or normalized late amplitude. At the pair level, *I*_PD_ peak amplitude was lower under the QX-314-containing intracellular condition across neuronal locations (main effect of intracellular solution: F(1,21) = 4.8, *p* = 0.04), with no solution-by-location interaction (F(1,21) = 0.003, *p* = 0.96). This main effect on peak amplitude did not reach significance after animal-level averaging (F(1,10) = 3.9, *p* = 0.08; Table A3). In contrast, animal-level reanalysis confirmed the solution-by-location interactions for zero-crossing time, peak latency, and normalized late amplitude (Appendix B, Figure A1C; Table A3). Together, these findings support a postsynaptic QX-314-sensitive contribution to the regional kinetic phenotype. Because QX-314 is not selective for GABA_B_ receptor-activated conductances, these results complement, but do not by themselves identify, the postsynaptic component implicated by the network-level CGP-55845 experiments.

### 2.5. No Difference in Gabbr1 or Gabbr2 mRNA Abundance Was Detected Between the Microgyrus and Contralateral Cortex

The regional differences in post-discharge outward-current kinetics, together with the pharmacological evidence for a GABA_B_-dependent contribution, raised the possibility that GABA_B_ receptor-subunit transcript abundance might differ in microgyral tissue. We therefore quantified *Gabbr1* and *Gabbr2* mRNA by RT-qPCR in paired samples from the microgyrus and the corresponding contralateral cortex of six FFL animals.

**Figure 7:**
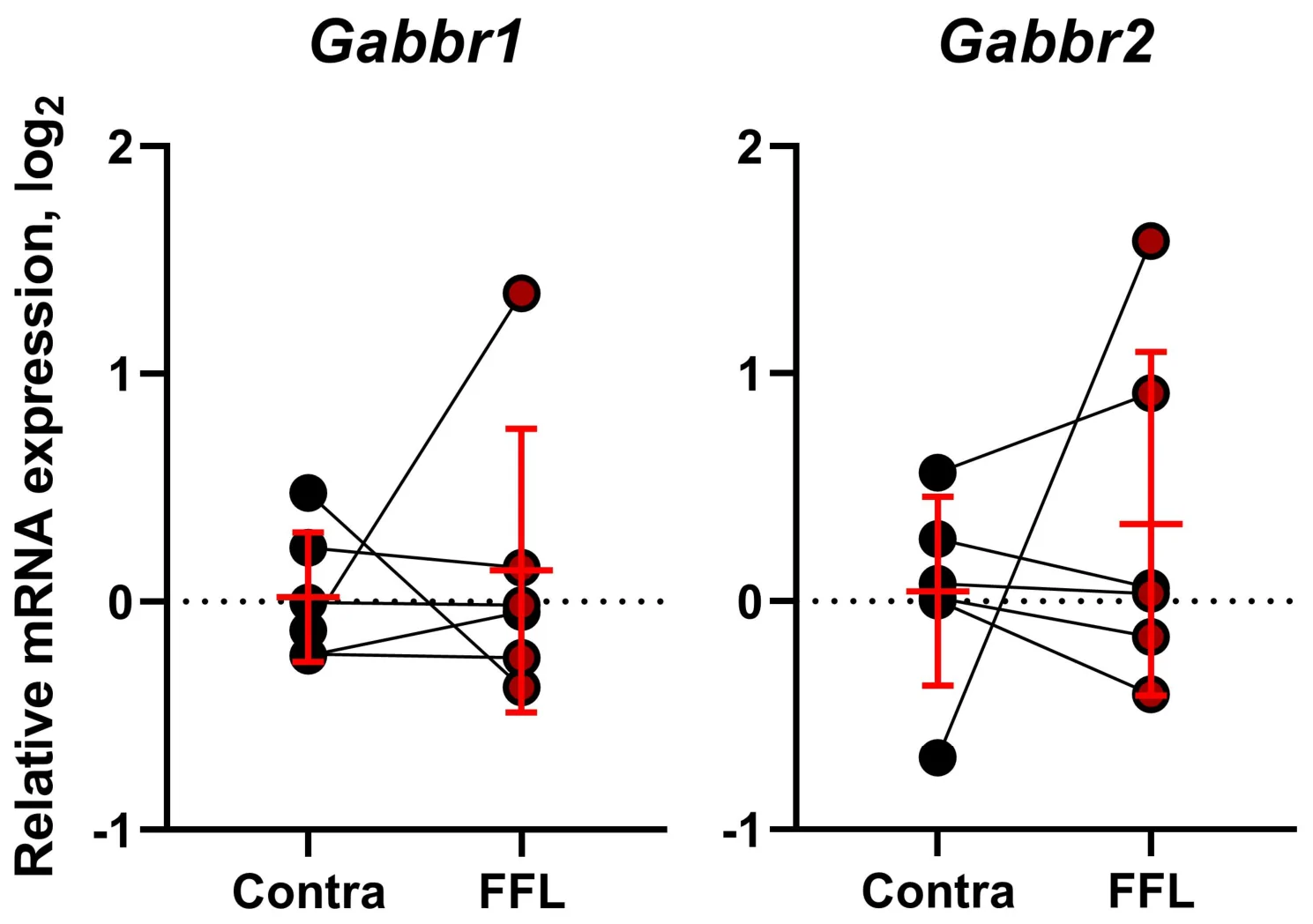
*Gabbr1* and *Gabbr2* mRNA abundance in paired microgyral and contralateral cortical samples. Relative mRNA abundance of *Gabbr1* and *Gabbr2* in microgyral tissue from the lesion-bearing hemisphere (FFL) and the corresponding contralateral cortex (Contra). Each line connects samples obtained from the same animal. Target-gene abundance was normalized to the reference genes Ppia, Pgk1, and Rpl13a and is presented on a log_2_ scale relative to the mean value in the matched contralateral samples. All six complete sample pairs were included (N = 6 animals). Red lines and error bars indicate mean ± SD. Paired *t*-test; *Gabbr1*: *p* = 0.72; *Gabbr2*: *p* = 0.50.

Paired *t*-tests detected no difference between the microgyrus and contralateral cortex in *Gabbr1* mRNA abundance (t(5) = 0.38, *p* = 0.72) or *Gabbr2* mRNA abundance (t(5) = 0.73, *p* = 0.50; Figure 7). Thus, no detectable microgyrus–contralateral difference in *Gabbr1* or *Gabbr2* mRNA abundance was observed. Because PMZ tissue was not analyzed by RT-qPCR, these measurements do not test whether transcript abundance differs between the two ipsilateral regions compared electrophysiologically.

## 3. Discussion

The principal finding of this study is that epileptiform discharges terminated earlier within the microgyrus than in the adjacent PMZ. This spatial difference was not accompanied by a detectable reduction in peak discharge-associated inward current or by faster decay of the measured extracellular K^+^ transient, arguing against these measured parameters as sufficient explanations. In separate voltage-clamp recordings, the regional termination phenotype was paralleled by an earlier transition from discharge-associated inward current to the post-discharge outward current (*I*_PD_), a shorter *I*_PD_ peak latency, and a smaller normalized late component in microgyral neurons. Because *I*_PD_ kinetics and discharge duration were measured in separate experimental configurations, this parallel regional pattern does not demonstrate a direct causal relationship. At the network level, GABA_B_ receptor blockade with CGP-55845 prolonged epileptiform discharges and eliminated the detectable regional difference in normalized post-peak current decay. At the cellular level, intracellular QX-314 eliminated detectable regional differences in *I*_PD_ kinetics. Together, these convergent findings support a postsynaptic GABA_B_-dependent contribution to the regional termination phenotype without establishing it as the sole cause of the shorter microgyral discharge.

The FFL cortex is spatially and functionally heterogeneous. In classical field-potential studies, evoked interictal-like activity was most readily generated in a focal paramicrogyral zone adjacent to, rather than within, the microgyrus. This activity persisted after surgical separation of the paramicrogyral zone from the malformation, indicating that reorganized adjacent circuits could continue to generate epileptiform events after disconnection from the microgyrus [28]. The location of the lowest-threshold initiation zone shifted away from the microsulcus with age, and epileptiform activity could propagate between the PMZ and microgyrus in either direction. These observations show that the lesion-bearing cortex comprises interacting compartments whose contributions to discharge initiation and propagation depend on developmental stage and experimental conditions. In our previous low-Mg^2+^/gabazine study, discharges were detected slightly earlier within the microgyrus in most simultaneous recordings, although that analysis did not address their termination [13]. The present results extend this spatial heterogeneity from the generation and propagation of epileptiform activity to the mechanisms that constrain its duration.

Several straightforward explanations for the earlier discharge termination within the microgyrus were not supported by our measurements. Peak discharge-associated inward current did not differ between the microgyrus and PMZ, arguing against a simple reduction in peak inward drive as a sufficient explanation. This measure, however, does not capture the temporal distribution of excitation, recurrent synaptic recruitment, or total excitatory charge transfer during the discharge. The peak extracellular K^+^ transient was higher in the PMZ. This difference could reflect the longer duration or greater cumulative activity of the events in that region, could contribute to their persistence by shifting EK and reducing the outward K^+^ driving force, or could result from both processes. More importantly, the weighted decay time constant of the measured K^+^ transient did not differ between regions, providing no evidence that faster decay of the local extracellular K^+^ signal explains earlier termination in the microgyrus. These findings narrow the range of plausible explanations but do not exclude differences in spatial K^+^ gradients, intracellular ion dynamics, electrogenic transport, or other activity-dependent processes that were not directly measured [14,15,16,17]. Importantly, the regional differences in *I*_PD_ kinetics and discharge termination were measured in separate experimental configurations (voltage-clamp and current-clamp, respectively); therefore, their parallel regional pattern should be interpreted as supportive association rather than direct causal evidence.

Post-discharge membrane responses in this preparation were not unitary but reflected the interaction of depolarizing and hyperpolarizing processes. At resting or hyperpolarized membrane potentials, epileptiform discharges were followed predominantly by a slow afterdepolarization, whereas membrane depolarization revealed a slow afterhyperpolarization and voltage-clamp recordings at depolarized holding potentials revealed a net post-discharge outward current, *I*_PD_. The sADP persisted after the discharge-associated synaptic current had largely returned to baseline, arguing against late synaptic excitation as its sole source. Simultaneous [K^+^]_o_ and membrane-potential recordings showed a prolonged extracellular K^+^ elevation and a corresponding depolarizing shift in E_K_ with a time course similar to that of the sADP. These observations are consistent with activity-dependent K^+^ accumulation contributing to the sADP, reducing the K^+^ driving force, and partly obscuring hyperpolarizing feedback at resting membrane potentials. Accordingly, the *I*_PD_ zero-crossing time reflects the balance between the decay of discharge-associated inward current and the emergence of outward current and should not be interpreted as a direct measure of GABA_B_ receptor activation kinetics. However, this temporal association does not isolate causality, and *I*_PD_ should be regarded as a net current that may include several ionic and electrogenic components rather than as a pharmacologically isolated GABA_B_-mediated current. Identifying the GABA_B_-dependent contribution therefore required separate pharmacological experiments.

Bath application of CGP-55845 provided network-level evidence that endogenous GABA_B_ receptor signaling contributes to epileptiform discharge termination. The antagonist transformed brief stereotypical discharges into prolonged multi-peak events in both sham-operated and FFL slices, consistent with previous observations that GABA_B_ receptor blockade can potentiate or prolong epileptiform activity in cortical and hippocampal preparations [21,22]. A related cortical precedent was obtained in entorhinal cortex, where CGP-55845 prolonged spontaneous and evoked persistent activity and prevented layer 1 stimulation from triggering transitions to the quiescent state [29]. Although cortical Up states are not equivalent to epileptiform discharges, these findings demonstrate that endogenous GABA_B_ signaling can actively contribute to the termination of ongoing cortical network activity. Subsequent genetic separation of GABA_B1_ receptor isoforms suggested preferential involvement of presynaptic GABA_B1a_-containing receptors in spontaneous termination and postsynaptic GABA_B1b_-containing receptors in afferent-evoked termination [30]. Thus, the relative contribution of pre- and postsynaptic GABA_B_ receptors may depend on the manner in which network activity is generated and terminated.

This compartmental complexity is important for interpreting the present CGP-55845 experiments. Because bath-applied CGP-55845 acts at both pre- and postsynaptic receptors and profoundly alters the underlying network trajectory, currents recorded before and after blockade cannot be treated as differing only by a pharmacologically isolated postsynaptic GABA_B_ component. The most informative regional result was therefore the significant drug-by-location interaction in normalized current decay. Under baseline conditions, this decay was faster in the microgyrus than in the PMZ; CGP-55845 slowed it in the microgyrus, whereas no corresponding change was detected in the PMZ, and the regional difference was no longer observed. These results support a GABA_B_-dependent contribution to the faster post-peak current decay observed in the microgyrus while leaving the relative roles of pre- and postsynaptic receptors unresolved.

To assess a postsynaptic component more directly, we introduced QX-314 intracellularly rather than blocking GABA_B_ receptors throughout the network. Intracellular QX-314 has long been known to suppress the slow GABA_B_-associated IPSP in hippocampal pyramidal neurons [26], and intracellular QX-314 was subsequently shown to reduce baclofen-evoked postsynaptic outward currents in rat globus pallidus neurons [31]. These findings support its use as a probe for a postsynaptic QX-314-sensitive component. In the present study, the regional differences in *I*_PD_ kinetics were no longer detected with intracellular QX-314. A pair-level reduction in *I*_PD_ peak amplitude did not reach significance after animal-level averaging and is therefore not treated as a robust animal-level effect. Because the manipulation was confined to recorded neurons, the kinetic findings support a postsynaptic contribution and complement the network-level effects of CGP-55845.

However, QX-314 is not selective for GABA_B_ receptor-activated conductances. In addition to blocking voltage-gated Na^+^ currents, intracellular QX-314 can alter voltage-gated Ca^2+^ currents [32] and can directly inhibit GIRK channels [27]. The latter action does not make the loss of regional differences diagnostic of GIRK involvement, because postsynaptic GABA_B_ signaling can recruit different K^+^ effectors across neuronal populations; for example, neocortical pyramidal neurons can couple GABA_B_ receptors to two-pore-domain K^+^ channels [33]. Thus, the convergence of the CGP-55845 and intracellular QX-314 findings supports a postsynaptic GABA_B_-dependent contribution to the regional phenotype, but neither the identity of the effector K^+^ conductance nor the fraction of *I*_PD_ mediated by GABA_B_ receptors can be established from the present experiments.

The molecular basis of the regional kinetic phenotype remains unresolved. No detectable difference in *Gabbr1* or *Gabbr2* mRNA abundance was observed between microgyral and contralateral cortical tissue, but this comparison does not reproduce the microgyrus–PMZ comparison used for electrophysiology and therefore cannot exclude transcript differences between those ipsilateral regions. Nor does it exclude cell-type-specific transcriptional changes, altered receptor protein abundance, surface trafficking, subcellular localization, or other post-transcriptional mechanisms. One testable hypothesis for future work is altered composition or regulation of KCTD auxiliary subunits. KCTD12 can accelerate the rise of GABA_B_ receptor-evoked K^+^ currents and promote rapid desensitization through activity-dependent interaction with Gβγ and uncoupling from effector channels; KCTD12 can also increase receptor surface availability [34,35,36]. KCTD12/KCTD16 hetero-oligomers confer distinct kinetic properties, and GABA_B_2 phosphorylation can regulate KCTD12-dependent desensitization [37,38]. These observations make KCTD composition or regulation a plausible candidate for future investigation, but they do not establish it as the mechanism underlying the present phenotype. Regional differences in receptor–G-protein–effector organization, postsynaptic K^+^ conductances, or RGS-family regulation provide additional possibilities; RGS7/Gβ5, for example, can accelerate GABA_B_–GIRK current deactivation [39].

An additional limitation concerns the cellular equivalence of the two recording regions. The microgyrus lacks the normal six-layered organization, making direct correspondence between neurons within the malformation and layer 2/3 pyramidal neurons in the PMZ inherently uncertain. Previous intracellular studies recovered biocytin-filled upper-layer pyramidal neurons from structurally affected FFL cortex without obvious qualitative morphological differences from sham-operated cortex, supporting the persistence of pyramidal-like principal neurons in the affected region [40]. However, those recordings did not directly validate the identity of neurons located within the microgyrus. In the present study, cells were selected as putative pyramidal neurons based on pyramidal somatic morphology and, in most cases, a visible apical dendrite; regular-spiking behavior provided additional electrophysiological support in current-clamp experiments. Nevertheless, post hoc morphological reconstruction or molecular cell-type identification was not performed. Regional differences in the sampled principal-cell populations, dendritic architecture, electrotonic filtering, somatodendritic organization of receptor–effector complexes, or the quality of somatic voltage control could therefore contribute to the measured *I*_PD_ kinetics. The pharmacological findings support a postsynaptic, QX-314-sensitive contribution to the regional phenotype but do not fully distinguish molecular remodeling within comparable neuronal populations from preferential sampling of functionally distinct principal-cell subtypes.

Other mechanisms may also contribute to the regional $I_{PD}$ phenotype. Because gabazine blocks GABA_A_ receptor-mediated transmission but does not prevent GABA release or GABA_B_ receptor activation, regional differences in interneuron density, connectivity, or presynaptic GABA_B_ autoregulation could alter the timing and magnitude of GABA reaching pyramidal neurons. Presynaptic GABA_B_ autoreceptors can suppress GABA release from interneuron terminals [41,42]. Such upstream differences are not excluded by our experiments. However, the loss of detectable regional differences in the presence of intracellular QX-314 supports the involvement of a postsynaptic QX-314-sensitive component. Our data therefore do not determine whether the regional phenotype originates solely from postsynaptic signaling or also reflects differential recruitment of GABAergic inputs.

The accelerated feedback observed within the microgyrus may represent a spatially localized compensatory adaptation that limits the duration of pathological synchronization. This remains an interpretation rather than a demonstrated function. Previous FFL studies indicate that lesion-induced reorganization is not uniformly pro-excitatory: affected pyramidal neurons can exhibit reduced firing gain, whereas altered synaptic connectivity to layer 5 fast-spiking interneurons has been interpreted as potentially strengthening inhibitory output [8,40]. Because the present experiments examined epileptiform discharges in acute slices under low-Mg^2+^/gabazine conditions, the conclusions apply specifically to epileptiform discharge termination in this preparation and should not be generalized directly to spontaneous seizure termination in vivo. Overall, the results argue against viewing malformed cortex as a uniformly hyperexcitable substrate and support a postsynaptic GABA_B_-dependent contribution as one component of the spatially heterogeneous processes that constrain discharge duration within the lesion-bearing cortex.

## 4. Materials and Methods

### 4.1. Animals

All experimental procedures were performed on male Wistar rats. The study utilized two distinct cohorts of animals. The first cohort (N = 30; 18 with FFLs and 12 sham-operated controls) was used for electrophysiological recordings and extracellular potassium measurements at postnatal days 21–23 (P21–P23). The second cohort (N = 6 rats; all with FFLs) was utilized exclusively for reverse transcription quantitative polymerase chain reaction (RT-qPCR) analysis at P21. Animals were housed under standard laboratory conditions (12 h light/dark cycle, constant temperature) with ad libitum access to food and water. The experimental protocol adhered to Directive 2010/63/EU and was approved by the Bioethics Committee of the Sechenov Institute of Evolutionary Physiology and Biochemistry (permit number 1–17, 26 January 2023). Only males were included to maintain consistency with the preceding FFL electrophysiological dataset on which this study builds and to limit an additional source of biological heterogeneity in this mechanistic study. Sex dependence was not examined and therefore generalization of the findings to females requires separate testing. All surgical and experimental procedures were designed to minimize animal suffering and the number of animals used.

### 4.2. FFL Model

Neocortical microgyria was induced in newborn rats (P0) within the first 24 h of life, following a modified protocol originally described by Dvorak and Feit [3]. Pups were anesthetized by hypothermia by placing them directly on a wet ice surface (≈0 °C), without an insulating barrier, for 4–5 min until the withdrawal reflex to a tail pinch was absent. No additional peri- or postoperative pharmacological analgesia was administered under the approved neonatal protocol. A rostro-caudal skin incision (≈8 mm) was made to expose the skull. Cortical lesions were generated by applying a cylindrical copper probe (1 mm tip diameter), pre-cooled to −60 °C, directly onto the intact skull over the left frontoparietal region (approximately 2 mm posterior to bregma and 3 mm lateral to the midline) for 8 s. Three subsequent freeze lesions were applied at 1 mm intervals along the caudal axis. Following the procedure, the incision was sealed with tissue adhesive, and pups were rewarmed on a heating pad under an infrared lamp until spontaneous motor activity resumed, after which they were returned to the dam. Sham-operated controls underwent the same surgical and hypothermic procedure without application of the freezing probe.

### 4.3. Brain Slice Preparation

After induction of deep isoflurane anesthesia, P21–P23 animals were decapitated; brains were rapidly extracted and immediately submerged in ice-cold ACSF continuously saturated with carbogen (95% O_2_ and 5% CO_2_). The standard ACSF comprised (in mM): 126 NaCl, 2.5 KCl, 1.25 NaH_2_PO_4_, 1 MgSO_4_, 2 CaCl_2_, 24 NaHCO_3_, and 10 D-glucose. Coronal brain sections (350 µm in thickness) were obtained using a vibrating microtome (HM 650 V; Microm International GmbH, Walldorf, Germany). Immediately after sectioning, the slices were transferred to a holding chamber filled with oxygenated ACSF and allowed to recover for 30–60 min at 35 °C before being maintained at room temperature. For subsequent electrophysiological recordings, 1 to 3 slices per animal were utilized.

### 4.4. In Vitro Model of Epileptiform Activity

Epileptiform activity was induced by perfusing the brain slices with a pro-epileptic ACSF. This modified solution contained a reduced Mg^2+^ concentration and the GABA_A_ receptor antagonist SR-95531 (gabazine; Alomone Labs, Jerusalem, Israel). The complete composition of the pro-epileptic ACSF was (in mM): 125 NaCl, 3.5 KCl, 2 CaCl_2_, 24 NaHCO_3_, 1.25 NaH_2_PO_4_, 0.25 MgSO_4_, 10 D-glucose, and 0.02 gabazine. Perfusion with this solution reliably generated stereotypical epileptiform bursts lasting approximately 1 s. To avoid cumulative pharmacological or metabolic effects, each slice was exposed to the pro-epileptic solution only once.

### 4.5. Whole-Cell Patch-Clamp Recordings

Whole-cell patch-clamp recordings were acquired in a continuously perfused recording chamber (flow rate: 6 mL/min) maintained at 30 °C. Layer II/III pyramidal neurons were visualized using an upright microscope (Nikon FN1; Nikon, Tokyo, Japan) equipped with differential interference contrast (DIC) optics and a digital camera (Grasshopper 3 GS3-U3-23S6M-C; FLIR Integrated Imaging Solutions, Wilsonville, OR, USA). Neurons were targeted within the superficial microgyral cortex or layers 2/3 of the PMZ. Putative pyramidal neurons were selected based on pyramidal somatic morphology and, in most cases, a visible apical dendrite. In current-clamp experiments, regular-spiking firing behavior provided additional electrophysiological support for neuronal classification. Post hoc morphological reconstruction was not performed.

Electrophysiological signals were recorded using a MultiClamp 700B amplifier (Molecular Devices, Sunnyvale, CA, USA) and digitized via an NI USB-6343 data acquisition board (National Instruments, Austin, TX, USA). Data were low-pass filtered at 10 kHz, sampled at 30 kHz, and collected using WinWCP 5 software (University of Strathclyde, Glasgow, UK).

Recording pipettes (3–5 MΩ) were pulled from borosilicate glass capillaries (Sutter Instrument, Novato, CA, USA) using a P-1000 micropipette puller (Sutter Instrument). The majority of experiments utilized simultaneous dual whole-cell recordings, yielding 2 to 4 neuronal pairs per animal. The same access-resistance criterion was applied across recording regions and intracellular solutions: series resistance was <12 MΩ at the start of each included recording and recordings were excluded if series resistance increased by ≥30% during acquisition. Series-resistance compensation was not used.

Depending on the specific experimental requirements, four distinct intracellular solutions were employed:

- *Solution 1* (standard current-clamp, in mM): 140 K-gluconate, 10 NaCl, 10 HEPES, 4 Mg-ATP, 0.3 Na-GTP, and 0.25 EGTA.
- *Solution 2* (voltage-clamp with voltage-gated K^+^ channel blockers, in mM): 139 K-gluconate, 9 NaCl, 1 Na-gluconate, 1 4-aminopyridine, 0.5 TEA-Cl, 10 HEPES, 4 Mg-ATP, 0.3 Na-GTP, and 0.25 EGTA.
- *Solution 3* (Cs^+^-based voltage-clamp, in mM): 127 Cs-methanesulfonate, 10 NaCl, 10 HEPES, 6 QX-314, 5 EGTA, 4 Mg-ATP, and 0.3 Na-GTP.
- *Solution 4* (K^+^-based voltage-clamp with QX-314, in mM): 133 K-gluconate, 9 NaCl, 6 QX-314, 1 Na-gluconate, 1 4-aminopyridine, 0.5 TEA-Cl, 10 HEPES, 4 Mg-ATP, 0.3 Na-GTP, and 0.25 EGTA.

The pH of all solutions was adjusted to 7.25 using KOH (Solutions 1, 2, and 4) or CsOH (Solution 3).

QX-314 chloride was obtained from Alomone Labs (Jerusalem, Israel; cat. no. Q-150). Pharmacological agents were applied as follows: the GABA_B_ receptor antagonist CGP-55845 (3 µM; Alomone Labs, Jerusalem, Israel) was bath-applied from a DMSO stock solution (final DMSO concentration ≤ 0.1%), with a minimum wash-in period of 6 min. The GABA_B_ receptor agonist SKF-97541 (5 µM; Alomone Labs) was locally applied via a micropipette positioned near the recorded soma using brief positive pressure pulses. To evoke epileptiform events, extracellular stimulation was delivered to the cortical tissue via a bipolar twisted nichrome electrode (0.1 ms pulses, 250–350 µA) during continuous perfusion with the pro-epileptic ACSF.

### 4.6. Extracellular Potassium Ion Concentration Recording

Extracellular potassium concentration ([K^+^]_o_) was measured using monopolar K^+^-selective microelectrodes, following established protocol [43,44]. Briefly, borosilicate glass pipettes were silanized with hexamethyldisilazane vapor (Sigma-Aldrich, St. Louis, MO, USA) at 220 °C for 90 min, backfilled with 100 mM KCl, and their tips were loaded with a K^+^ sensor (Potassium Ionophore I, cocktail A; Sigma-Aldrich, cat. no. 99311) via gentle suction. Electrode potentials were recorded in zero-current clamp mode using a Multiclamp 700B amplifier (Molecular Devices, Sunnyvale, CA, USA). Only recordings from electrodes demonstrating stable calibration before and after the experiment were included in the analysis.

The [K^+^]_o_ at time *t* was calculated from the recorded voltage *V(t)* as follows:

(1) $${\left[K^{+}\right]}_{o}\left(t\right)=2.5e^{S*V(t)}$$

where the scaling factor *S* was empirically determined as 0.044 mV^−1^ (range: 0.043–0.045 mV^−1^) using a fast perfusion system (HSSE-2/3, ALA Scientific Instruments, Farmingdale, NY, USA).

Nernst equation was used in order to reconstitute the assumed K^+^ equilibrium potential *E*_K_ of the recorded neuron from the ${\left[K^{+}\right]}_{o}$:

(2) $$E_{K}=26.1*\ln\frac{{\left[K^{+}\right]}_{o}}{{\left[K^{+}\right]}_{i}}$$

where ${\left[K^{+}\right]}_{i}$ is the concentration of potassium ions in the pipette solution.

To quantify potassium clearance kinetics, the post-discharge [K^+^]_o_ decay (from 90% to 10% of the peak) was fitted with a bi-exponential function, and the weighted decay time constant (τ_weighted_) was calculated as:

(3) $$\tau_{weighted}=\frac{A_{1}*\tau_{1}+A_{2}*\tau_{2}}{A_{1}+A_{2}}$$

where τ_1_ and τ_2_ represent the time constants, and *A*_1_ and *A*_2_ denote the amplitudes of the respective decaying components.

### 4.7. Electrophysiological Data Analysis

Electrophysiological data were analyzed using custom scripts written in Wolfram Mathematica 12 (Wolfram Research, Champaign, IL, USA). To ensure the stabilization of network activity, the characteristics of spontaneous and evoked epileptiform discharges were assessed during a 20 to 40 min interval following the onset of perfusion with the pro-epileptic ACSF. For each recording, all eligible epileptiform events occurring within the 20–40 min analysis window were included in the analysis. Events were aligned by their onset—defined as the point of rapid depolarization in current-clamp mode or the initial current deflection in voltage-clamp mode—and averaged to obtain a single representative trace per neuron or neuronal pair. The number of events contributing to each average ranged from 5 to 20 per recording, depending on the discharge frequency in each slice.

The following event types were excluded from the analysis of individual discharge properties: high-frequency bursts (defined as clusters of 2–7 discharges occurring within a 2 to 5 s window without a return to baseline between events); prolonged seizure-like events (discharges lasting >5 s with complex, multi-peak morphology); and spreading depolarizations (identified by a sustained negative DC shift >20 mV in current-clamp or >1 nA in voltage-clamp, lasting >10 s and accompanied by prolonged suppression of spontaneous activity).

Discharge duration was defined as the time interval during which the membrane potential remained above −50 mV in current-clamp mode, or as the duration at 50% of the peak amplitude in voltage-clamp recordings. The normalized current decay slope was calculated by fitting a linear regression to the decay phase within a standardized post-peak window (70–220 ms after the initial peak) and dividing the resulting slope by the peak current amplitude. For the slow afterhyperpolarization current (*I*_PD_), the onset was defined as the zero-current crossing point, peak latency as the time from onset to maximum amplitude, and relative decay as the normalized amplitude measured 10 s post-onset.

### 4.8. Reverse Transcription Followed by Quantitative Polymerase Chain Reaction (RT-qPCR)

At P21, animals from the second cohort were deeply anesthetized with isoflurane before decapitation. Using a cryostat microtome (OTF5000, Bright Instruments, Luton, UK), the ipsilateral cortical area bearing the microgyrus (FFL) and the corresponding contralateral cortical region were carefully dissected from each of the six animals. Total RNA isolation, DNase treatment, cDNA synthesis, and subsequent RT-qPCR were performed according to our previously established protocols [45]. Briefly, 1 µg of total RNA was reverse-transcribed, and qPCR was conducted using TaqM polymerase (Alkor Bio, Saint Petersburg, Russia) on a CFX384 Touch Real-Time PCR Detection System (Bio-Rad, Hercules, CA, USA). The expression levels of the target genes (*Gabbr1* and *Gabbr2*) were normalized to the geometric mean of the most stable reference genes (Ppia, Pgk1, and Rpl13a). Reference-gene stability was evaluated as described previously [46]. Relative gene expression was calculated using the 2^−ΔΔCt^ method [47]. Primer and probe sequences are provided in Table A2.

Primers for *Gabbr1* and *Gabbr2* were designed using Primer-BLAST (https://www.ncbi.nlm.nih.gov/tools/primer-blast/, accessed on 24 September 2025) based on NCBI RefSeq cDNA sequences. Probes were designed with Primer3Plus (https://www.primer3plus.com/index.html, accessed on 24 September 2025). Primer parameters: amplicon 50–80 nt, Tm 58–63 °C, ΔTm ≤ 3 °C. Probe specifications: 18–27 nt, 20–80% GC, no 5′ G, Tm 5–10 °C above primers. Reaction efficiency, validated by serial dilution method [48], was 90–100%.

### 4.9. Statistical Analysis

Statistical analyses were performed using GraphPad Prism 9 (GraphPad Software, San Diego, CA, USA). Outliers were identified using Dixon’s Q-test (95% confidence level); no outliers were detected in any of the datasets used for the main analyses presented in this study. Data normality was assessed using the Shapiro–Wilk test, and variance homogeneity was evaluated using Levene’s test. Depending on the data distribution, comparisons between two groups were performed using the paired or unpaired Student’s *t*-test, or Welch’s *t*-test (when variances were unequal). For non-normally distributed paired data, the Wilcoxon signed-rank test was used. Multiple comparisons were analyzed using two-way repeated-measures ANOVA or mixed-model ANOVA, followed by Sidak’s post hoc test. Data are presented as mean ± standard deviation (SD). The sample size (*n*) represents the number of neurons or neuronal pairs for dual recordings. N represents the number of animals. Statistical significance was defined as *p* < 0.05.

To assess whether clustering of multiple recordings within animals affected the principal inferences, comparisons central to the main conclusions were additionally evaluated using an animal-level summary approach. For each animal, data from all recorded cells or neuronal pairs contributing to a given analysis were averaged, and the resulting animal-level values were analyzed with the corresponding statistical test. These supplementary analyses are presented in Appendix B and are referenced in the Results where relevant.

## 5. Conclusions

In the rat FFL model, epileptiform discharges terminated earlier within the microgyrus than in the adjacent PMZ. This regional difference was not accompanied by detectable differences in peak discharge-associated inward current or in the weighted decay time constant of the extracellular K^+^ transient. GABA_B_ receptor blockade preferentially disrupted the faster post-peak current decay in the microgyrus, whereas intracellular QX-314 eliminated detectable regional differences in post-discharge outward-current kinetics. Together, these convergent findings support a postsynaptic GABA_B_-dependent contribution to accelerated epileptiform discharge termination within the microgyrus but do not establish it as the sole cause of the regional duration difference or identify the effector K^+^ conductance. No detectable difference in *Gabbr1* or *Gabbr2* mRNA abundance was observed between the microgyrus and contralateral cortex; because PMZ tissue was not analyzed, the underlying molecular basis of the microgyrus–PMZ electrophysiological phenotype remains unresolved.

## Data Availability

The data presented in this study are available on request from the corresponding author.

## Abbreviations

The following abbreviations are used in this manuscript:

**Abbreviation**	**Definition**
ACSF	artificial cerebrospinal fluid
ANOVA	analysis of variance
CC	current-clamp
DMSO	dimethyl sulfoxide
FCD	focal cortical dysplasia
FFL	focal freeze lesion
GIRK	G protein-coupled inwardly rectifying potassium
I_K_	intermediate-conductance
*I*_PD_	slow post-discharge outward current
mEPSC	miniature excitatory postsynaptic current
PMZ	paramicrogyral zone
qPCR	quantitative polymerase chain reaction
RGS	regulator of G-protein signaling
RM ANOVA	repeated measures analysis of variance
RT-qPCR	reverse transcription quantitative polymerase chain reaction
sADP	slow afterdepolarization
sAHP	slow afterhyperpolarization
SD	standard deviation
SK	small conductance
SLE	seizure-like event
VC	voltage-clamp

## Appendix A

**Table A1:** The results of the mixed model ANOVA analysis of *I*_PD_ characteristics.

	Factors		
Measured Parameter	QX-314	Location	QX-314 × Location
*I*_PD_ peak	F(1,21) = 4.8, *p* = 0.04	F(1,21) = 4.0, *p* = 0.06	F(1,21) = 0.003, *p* = 0.96
*I*_PD_ late normalized amplitude	F(1,21) = 3.4, *p* = 0.08	F(1,21) = 2.0, *p* = 0.18	F(1,21) = 5.4, *p* = 0.030
*I*_PD_ onset	F(1,20) = 27.8, *p* < 0.0001	F(1,20) = 1.2, *p* = 0.28	F(1,20) = 6.6, *p* = 0.019
*I*_PD_ latency	F(1,21) = 9.4, *p* = 0.006	F(1,21) = 10.4, *p* = 0.004	F(1,21) = 5.5, *p* = 0.029

**Table A2:** Primers and probes used in RT-qPCR.

Gene Symbol	Sequences of Primer and Probe (5′ → 3′)	Final Concentration, nM	References
*Actb* NM_031144	TGTCACCAACTGGGACGATA GGGGTGTTGAAGGTCTCAAA FAM-CGTGTGGCCCCTGAGGAGCAC-BHQ1	200 200 200	[49] (primers) [50] (probe)
*Gapdh* NM_017008	TGCACCACCAACTGCTTAG GGATGCAGGGATGATGTTC R6G-ATCACGCCACAGCTTTCCAGAGGG-BHQ2	200 200 100	[51]
*B2m* NM_012512	TGCCATTCAGAAAACTCCCC GAGGAAGTTGGGCTTCCCATT ROX-ATTCAAGTGTACTCTCGCCATCCACCG-BHQ1	200 200 100	[52]
*Rpl13a* NM_173340	GGATCCCTCCACCCTATGACA CTGGTACTTCCACCCGACCTC FAM-CTGCCCTCAAGGTTGTGCGGCT-BHQ1	200 200 100	[53] (primers) [50] (probe)
*Sdha* NM_130428	AGACGTTTGACAGGGGAATG TCATCAATCCGCACCTTGTA R6G-ACCTGGTGGAGACGCTGGAGCT-BHQ2	200 200 100	[54] (primers) [50] (probe)
*Ppia* NM_017101	AGGATTCATGTGCCAGGGTG CTCAGTCTTGGCAGTGCAGA ROX-CACGCCATAATGGCACTGGTGGCA-BHQ1	200 200 100	[55]
*Hprt1* NM_012583	TCCTCAGACCGCTTTTCCCGC TCATCATCACTAATCACGACGCTGG FAM-CCGACCGGTTCTGTCATGTCGACCCT-BHQ1	200 200 100	[56] (primers) [50] (probe)
*Pgk1* NM_053291	ATGCAAAGACTGGCCAAGCTAC AGCCACAGCCTCAGCATATTTC R6G-TGCTGGCTGGATGGGCTTGGA-BHQ2	200 200 100	[57] (primers) [50] (probe)
*Ywhaz* NM_013011	GATGAAGCCATTGCTGAACTTG GTCTCCTTGGGTATCCGATGTC ROX-TGAAGAGTCGTACAAAGACAGCACGC-BHQ1	200 200 100	[57] (primers) [50] (probe)
*Gabbr1* NM_031028.3	TGTAGTCAGGGCCAGTGGAG ATACTGCACGCCGTTCTGAG HEX-AGCCCCACTGCCAGGTGAATCGAAC-BHQ2	200 200 100	This work
*Gabbr2* NM_031802.2	CCTGAAGCACTTCCGTTGGC CAGGTCATTCCTCACCTCGGA ROX-TGTGGGCACACTCACGCAGGACGTG-BHQ2	200 200 200	This work

## Appendix B

Animal-level reanalysis of findings central to the main conclusions. For each analysis shown below, measurements from individual neurons or neuronal pairs were first averaged within each animal and statistical comparisons were then performed on these animal-level values. The principal regional effects and solution-by-location interactions underlying the conclusions were retained. The pair-level main effect of intracellular solution on *I*_PD_ peak amplitude did not reach significance after animal-level averaging (*p* = 0.08; Table A3).

**Table A3:** The results of the mixed model ANOVA analysis of *I*_PD_ characteristics (animal-level averages).

	Factors		
Measured Parameter	QX-314	Location	QX-314 × Location
IPD peak	F(1,10) = 3.9, *p* = 0.08	F(1,10) =1.1, *p* = 0.32	F(1,10) = 0.3, *p* = 0.58
IPD late normalized amplitude	F(1,10) = 1.8, *p* = 0.21	F(1,10) = 2.7, *p* = 0.13	F(1,10) = 6.2, *p* = 0.032
IPD onset	F(1,10) = 24.7, *p* = 0.0006	F(1,10) = 0.7, *p* = 0.41	F(1,10) = 6.5, *p* = 0.029
IPD latency	F(1,10) = 9.6, *p* = 0.011	F(1,10) = 8.5, *p* = 0.015	F(1,10) = 5.4, *p* = 0.042

## References

[1] Leventer R.J., Guerrini R., Dobyns W.B. (2008). Malformations of Cortical Development and Epilepsy. Dialogues Clin. Neurosci..

[2] Barkovich A.J., Guerrini R., Kuzniecky R.I., Jackson G.D., Dobyns W.B. (2012). A Developmental and Genetic Classification for Malformations of Cortical Development: Update 2012. Brain.

[3] Dvorák K., Feit J. (1977). Migration of Neuroblasts through Partial Necrosis of the Cerebral Cortex in Newborn Rats-Contribution to the Problems of Morphological Development and Developmental Period of Cerebral Microgyria. Histological and Autoradiographical Study. Acta Neuropathol..

[4] Luhmann H.J. (2016). Models of Cortical Malformation--Chemical and Physical. J. Neurosci. Methods.

[5] Jacobs K.M., Gutnick M.J., Prince D.A. (1996). Hyperexcitability in a Model of Cortical Maldevelopment. Cereb. Cortex.

[6] Jacobs K.M., Kharazia V.N., Prince D.A. (1999). Mechanisms Underlying Epileptogenesis in Cortical Malformations. Epilepsy Res..

[7] Brill J., Huguenard J.R. (2010). Enhanced Infragranular and Supragranular Synaptic Input onto Layer 5 Pyramidal Neurons in a Rat Model of Cortical Dysplasia. Cereb. Cortex.

[8] Jin X., Jiang K., Prince D.A. (2014). Excitatory and Inhibitory Synaptic Connectivity to Layer V Fast-Spiking Interneurons in the Freeze Lesion Model of Cortical Microgyria. J. Neurophysiol..

[9] Zilles K., Qü M., Schleicher A., Luhmann H.J. (1998). Characterization of Neuronal Migration Disorders in Neocortical Structures: Quantitative Receptor Autoradiography of Ionotropic Glutamate, GABA_A_ and GABA_B_ Receptors. Eur. J. Neurosci..

[10] Hagemann G., Kluska M.M., Redecker C., Luhmann H.J., Witte O.W. (2003). Distribution of Glutamate Receptor Subunits in Experimentally Induced Cortical Malformations. Neuroscience.

[11] Redecker C., Luhmann H.J., Hagemann G., Fritschy J.-M., Witte O.W. (2000). Differential Downregulation of GABA_A_ Receptor Subunits in Widespread Brain Regions in the Freeze-Lesion Model of Focal Cortical Malformations. J. Neurosci..

[12] Shimizu-Okabe C., Okabe A., Kilb W., Sato K., Luhmann H.J., Fukuda A. (2007). Changes in the Expression of Cation-Cl−Cotransporters, NKCC1 and KCC2, during Cortical Malformation Induced by Neonatal Freeze-Lesion. Neurosci. Res..

[13] Malkin S.L., Amakhin D.V., Soboleva E.B., Postnikova T.Y., Zaitsev A.V. (2025). Synaptic Dysregulation Drives Hyperexcitability in Pyramidal Neurons Surrounding Freeze-Induced Neocortical Malformations in Rats. Int. J. Mol. Sci..

[14] Krishnan G.P., Bazhenov M. (2011). Ionic Dynamics Mediate Spontaneous Termination of Seizures and Postictal Depression State. J. Neurosci..

[15] Krishnan G.P., Filatov G., Shilnikov A., Bazhenov M. (2015). Electrogenic Properties of the Na^+^/K^+^ ATPase Control Transitions between Normal and Pathological Brain States. J. Neurophysiol..

[16] Gentiletti D., de Curtis M., Gnatkovsky V., Suffczynski P. (2022). Focal Seizures Are Organized by Feedback between Neural Activity and Ion Concentration Changes. Elife.

[17] Amakhin D.V., Sinyak D.S., Soboleva E.B., Zaitsev A.V. (2025). HCN Channels Promote Na/K-ATPase Activity during Slow Afterhyperpolarization after Seizure-like Events in vitro. J. Physiol..

[18] Bettler B., Kaupmann K., Mosbacher J., Gassmann M. (2004). Molecular Structure and Physiological Functions of GABA(B) Receptors. Physiol. Rev..

[19] Couve A., Moss S.J., Pangalos M.N. (2000). GABAB Receptors: A New Paradigm in G Protein Signaling. Mol. Cell. Neurosci..

[20] Lüscher C., Jan L.Y., Stoffel M., Malenka R.C., Nicoll R.A. (1997). G Protein-Coupled Inwardly Rectifying K^+^ Channels (GIRKs) Mediate Postsynaptic but Not Presynaptic Transmitter Actions in Hippocampal Neurons. Neuron.

[21] Karlsson G., Kolb C., Hausdorf A., Portet C., Schmutz M., Olpe H.R. (1992). GABAB Receptors in Various in Vitro and in Vivo Models of Epilepsy: A Study with the GABAB Receptor Blocker CGP 35348. Neuroscience.

[22] Sutor B., Luhmann H.J. (1998). Involvement of GABA(B) Receptors in Convulsant-Induced Epileptiform Activity in Rat Neocortex In Vitro. Eur. J. Neurosci..

[23] Smirnova E.Y., Chizhov A.V., Zaitsev A.V. (2020). Presynaptic GABAB Receptors Underlie the Antiepileptic Effect of Low-Frequency Electrical Stimulation in the 4-Aminopyridine Model of Epilepsy in Brain Slices of Young Rats. Brain Stimul..

[24] Avoli M., Lévesque M. (2022). GABAB Receptors: Are They Missing in Action in Focal Epilepsy Research?. Curr. Neuropharmacol..

[25] Gulledge A.T., Dasari S., Onoue K., Stephens E.K., Hasse J.M., Avesar D. (2013). A Sodium-Pump-Mediated Afterhyperpolarization in Pyramidal Neurons. J. Neurosci..

[26] Nathan T., Jensen M.S., Lambert J.D. (1990). The Slow Inhibitory Postsynaptic Potential in Rat Hippocampal CA1 Neurones Is Blocked by Intracellular Injection of QX-314. Neurosci. Lett..

[27] Slesinger P.A. (2001). Ion Selectivity Filter Regulates Local Anesthetic Inhibition of G-Protein-Gated Inwardly Rectifying K^+^ Channels. Biophys. J..

[28] Jacobs K.M., Hwang B.J., Prince D.A. (1999). Focal Epileptogenesis in a Rat Model of Polymicrogyria. J. Neurophysiol..

[29] Mann E.O., Kohl M.M., Paulsen O. (2009). Distinct Roles of GABA(A) and GABA(B) Receptors in Balancing and Terminating Persistent Cortical Activity. J. Neurosci..

[30] Craig M.T., Mayne E.W., Bettler B., Paulsen O., McBain C.J. (2013). Distinct Roles of GABAB1a- and GABAB1b-Containing GABAB Receptors in Spontaneous and Evoked Termination of Persistent Cortical Activity. J. Physiol..

[31] Kaneda K., Kita H. (2005). Synaptically Released GABA Activates Both Pre- and Postsynaptic GABA(B) Receptors in the Rat Globus Pallidus. J. Neurophysiol..

[32] Talbot M.J., Sayer R.J. (1996). Intracellular QX-314 Inhibits Calcium Currents in Hippocampal CA1 Pyramidal Neurons. J. Neurophysiol..

[33] Breton J.-D., Stuart G.J. (2017). GABAB Receptors in Neocortical and Hippocampal Pyramidal Neurons Are Coupled to Different Potassium Channels. Eur. J. Neurosci..

[34] Ivankova K., Turecek R., Fritzius T., Seddik R., Prezeau L., Comps-Agrar L., Pin J.-P., Fakler B., Besseyrias V., Gassmann M. (2013). Up-Regulation of GABA(B) Receptor Signaling by Constitutive Assembly with the K^+^ Channel Tetramerization Domain-Containing Protein 12 (KCTD12). J. Biol. Chem..

[35] Turecek R., Schwenk J., Fritzius T., Ivankova K., Zolles G., Adelfinger L., Jacquier V., Besseyrias V., Gassmann M., Schulte U. (2014). Auxiliary GABAB Receptor Subunits Uncouple G Protein βγ Subunits from Effector Channels to Induce Desensitization. Neuron.

[36] Li M., Milligan C.J., Wang H., Walker A., Churilov L., Lawrence A.J., Reid C.A., Hopkins S.C., Petrou S. (2017). KCTD12 Modulation of GABA(B) Receptor Function. Pharmacol. Res. Perspect..

[37] Adelfinger L., Turecek R., Ivankova K., Jensen A.A., Moss S.J., Gassmann M., Bettler B. (2014). GABAB Receptor Phosphorylation Regulates KCTD12-Induced K^+^ Current Desensitization. Biochem. Pharmacol..

[38] Fritzius T., Turecek R., Seddik R., Kobayashi H., Tiao J., Rem P.D., Metz M., Kralikova M., Bouvier M., Gassmann M. (2017). KCTD Hetero-Oligomers Confer Unique Kinetic Properties on Hippocampal GABA_B_ Receptor-Induced K^+^ Currents. J. Neurosci..

[39] Fajardo-Serrano A., Wydeven N., Young D., Watanabe M., Shigemoto R., Martemyanov K.A., Wickman K., Luján R. (2013). Association of Rgs7/Gβ5 Complexes with Girk Channels and GABA_B_ Receptors in Hippocampal CA1 Pyramidal Neurons. Hippocampus.

[40] Luhmann H.J., Karpuk N., Qü M., Zilles K. (1998). Characterization of Neuronal Migration Disorders in Neocortical Structures. II. Intracellular in Vitro Recordings. J. Neurophysiol..

[41] Kobayashi M., Takei H., Yamamoto K., Hatanaka H., Koshikawa N. (2012). Kinetics of GABAB Autoreceptor-Mediated Suppression of GABA Release in Rat Insular Cortex. J. Neurophysiol..

[42] Booker S.A., Harada H., Elgueta C., Bank J., Bartos M., Kulik A., Vida I. (2020). Presynaptic GABAB Receptors Functionally Uncouple Somatostatin Interneurons from the Active Hippocampal Network. Elife.

[43] Octeau J.C., Faas G., Mody I., Khakh B.S. (2018). Making, Testing, and Using Potassium Ion Selective Microelectrodes in Tissue Slices of Adult Brain. J. Vis. Exp..

[44] Chizhov A.V., Amakhin D.V., Smirnova E.Y., Zaitsev A.V. (2022). Ictal Wavefront Propagation in Slices and Simulations with Conductance-Based Refractory Density Model. PLoS Comput. Biol..

[45] Kovalenko A.A., Zakharova M.V., Schwarz A.P., Dyomina A.V., Zubareva O.E., Zaitsev A.V. (2022). Changes in Metabotropic Glutamate Receptor Gene Expression in Rat Brain in a Lithium–Pilocarpine Model of Temporal Lobe Epilepsy. Int. J. Mol. Sci..

[46] Schwarz A.P., Kovalenko A.A., Malygina D.A., Postnikova T.Y., Zubareva O.E., Zaitsev A.V. (2020). Reference Gene Validation in the Brain Regions of Young Rats after Pentylenetetrazole-Induced Seizures. Biomedicines.

[47] Livak K.J., Schmittgen T.D. (2001). Analysis of Relative Gene Expression Data Using Real-Time Quantitative PCR and the 2^−ΔΔCT^ Method. Methods.

[48] Svec D., Tichopad A., Novosadova V., Pfaffl M.W., Kubista M. (2015). How Good Is a PCR Efficiency Estimate: Recommendations for Precise and Robust QPCR Efficiency Assessments. Biomol. Detect. Quantif..

[49] Bonefeld B.E., Elfving B., Wegener G. (2008). Reference Genes for Normalization: A Study of Rat Brain Tissue. Synapse.

[50] Schwarz A.P., Malygina D.A., Kovalenko A.A., Trofimov A.N., Zaitsev A.V. (2020). Multiplex QPCR Assay for Assessment of Reference Gene Expression Stability in Rat Tissues/Samples. Mol. Cell. Probes.

[51] Lin W., Burks C.A., Hansen D.R., Kinnamon S.C., Gilbertson T.A. (2004). Taste Receptor Cells Express PH-Sensitive Leak K^+^ Channels. J. Neurophysiol..

[52] Yamaguchi M., Yamauchi A., Nishimura M., Ueda N., Naito S. (2005). Soybean Oil Fat Emulsion Prevents Cytochrome P450 MRNA Down-Regulation Induced by Fat-Free Overdose Total Parenteral Nutrition in Infant Rats. Biol. Pharm. Bull..

[53] Swijsen A., Nelissen K., Janssen D., Rigo J.M., Hoogland G. (2012). Validation of Reference Genes for Quantitative Real-Time PCR Studies in the Dentate Gyrus after Experimental Febrile Seizures. BMC Res. Notes.

[54] Pohjanvirta R., Niittynen M., Lindén J., Boutros P.C., Moffat I.D., Okey A.B. (2006). Evaluation of Various Housekeeping Genes for Their Applicability for Normalization of MRNA Expression in Dioxin-Treated Rats. Chem. Biol. Interact..

[55] Malkin S.L., Amakhin D.V., Veniaminova E.A., Kim K.K., Zubareva O.E., Magazanik L.G., Zaitsev A. (2016). V Changes of AMPA Receptor Properties in the Neocortex and Hippocampus Following Pilocarpine-Induced Status Epilepticus in Rats. Neuroscience.

[56] Cook N.L., Vink R., Donkin J.J., van den Heuvel C. (2009). Validation of Reference Genes for Normalization of Real-Time Quantitative RT-PCR Data in Traumatic Brain Injury. J. Neurosci. Res..

[57] Langnaese K., John R., Schweizer H., Ebmeyer U., Keilhoff G. (2008). Selection of Reference Genes for Quantitative Real-Time PCR in a Rat Asphyxial Cardiac Arrest Model. BMC Mol. Biol..

